# Effects of the concentration and nature of total dissolved solids in drinking water on feed intake, nutrient digestion, energy balance, methane emission, ruminal fermentation, and blood constituents in different breeds of young goats and hair sheep

**DOI:** 10.1016/j.aninu.2023.10.002

**Published:** 2023-10-30

**Authors:** Amlan Kumar Patra, Luana Paula dos Santos Ribeiro, Hirut Yirga, Adekayode O. Sonibare, Ahmed R. Askar, Ali H. Hussein, Ryszard Puchala, Arthur Louis Goetsch

**Affiliations:** a American Institute for Goat Research, Langston University, Langston, Oklahoma, 73050, USA; b Animal and Poultry Nutrition Department, Desert Research Center, El-Matareya, Cairo, 11753, Egypt; c Agriculture, Food and Natural Resources, Eastern New Mexico University, Portales, New Mexico, 88130, USA

**Keywords:** Goats, Hair sheep, Nutrient utilization, Ruminal fermentation, Salinity, Water quality

## Abstract

Understanding how different livestock species and breeds respond to consumption of brackish water could improve usage of this resource. Therefore, Angora, Boer, and Spanish goat doelings and Dorper, Katahdin, and St. Croix ewe lambs (6 animals per animal type [AT]; initial age = 296 ± 2.1 days) consuming water with varying concentrations of minerals of a natural brackish water source (BR) and sodium chloride (NaCl; SL) were used to determine effects on water and feed intake, nutrient digestion, heat energy, methane emission, ruminal fluid conditions, and blood constituent concentrations. There were 6 simultaneous 6 (water treatments [WT]) × 6 (AT) Latin squares with 3-wk periods. The WT were fresh (FR), BR alone (100-BR), a similar total dissolved solids (TDS) concentration as 100-BR via NaCl addition to FR (100-SL), BR with concentrations of all minerals increased by approximately 50% (150-BR), a similar TDS level as 150-BR by NaCl addition to FR (150-SL), and a similar 150 TDS level achieved by addition of a 1:1 mixture of BR minerals and NaCl to 100-BR (150-BR/SL). Concentrations (mg/kg) in BR were 4928 TDS, 85.9 bicarbonate, 224.9 calcium, 1175 chloride, 60.5 magnesium, 4.59 potassium, 1387 sodium, 1962 sulfate, and 8.3 boron, and TDS in other WT were 209, 5684, 7508, 8309, and 7319 mg/kg for FR, 100-SL, 150-BR, 150-SL, and 150-BR/SL, respectively. There were very few significant effects of WT or AT × WT interactions, although AT had numerous effects. Water intake was affected by AT (*P* = 0.02) and WT (*P* = 0.04), with greater water intake for 150-SL than for FR, 100-BR, 100-SL, and 150-BR. Dry matter intake among AT was lowest (*P* < 0.05) for Angora. Digestion of organic matter and neutral detergent fiber and heat energy differed among AT (*P* < 0.05), but nitrogen digestion and ruminal methane emission were similar among AT. Blood aldosterone concentration was higher (*P* < 0.05) for FR than for other WT. In conclusion, all AT seemed resilient to these WT regardless of mineral source and concentrations, with TDS less than 8300 mg/kg, which did not influence nutrient utilization, ruminal fermentation, energy balance, or blood constituent levels.

## 1 Introduction

Global livestock production is responsible for the use of approximately 30% of total agricultural water (Mekonnen and Hoekstra, 2012). The quality of water can have a marked impact on the level and efficiency of production by livestock (Abdelsattar et al., 2020; Wagner and Engle, 2021). Because of factors such as low rainfall and use of fresh water for other purposes, ruminants often drink brackish or saline water, particularly in semiarid and arid regions of the world (Abdelsattar et al., 2020). Furthermore, with climate change and greater competition for water resources between human, livestock, agriculture, and industrial uses and considering the high cost of desalinization (Mekonnen and Hoekstra, 2012; Ran et al., 2016), it is anticipated that livestock will increasingly rely on brackish/saline water in the future. However, drinking saline and brackish water may have adverse effects, particularly on feed intake and digestibility (Petersen et al., 2015; Sharma et al., 2017; Yousfi and Salem, 2017), reproduction, and health (Mittal and Ghosh, 1983; Patterson et al., 2004; Wagner and Engle, 2021). Although, in some cases animal performance has not been markedly affected (Alves et al., 2017; de Araújo et al., 2019; Yousfi et al., 2016). Moura et al. (2016) reported that water with a total dissolved solids (TDS) level between 5120 and 7040 mg/L can be used for ruminants, as also proposed by McGregor (2004) for young sheep and goats. In beef cattle, TDS levels less than 3000 mg/L are generally regarded as safe (Wagner and Engle, 2021). In addition, Moura et al. (2016) proposed that levels above 7040 mg/L pose a considerable risk to very young, pregnant, and lactating animals, with levels above 10,240 mg/L unsuitable for any species of livestock. However, the tolerance to drinking saline water can vary among animal types, species, TDS concentration, duration of consumption (Abou Hussien et al., 1994a; Saul and Flinn, 1985; Tsukahara et al., 2016; Yirga et al., 2018), physiological state (Moehlenpah et al., 2021; Yirga et al., 2018), and specific minerals in the water as well as the diet (Mdletshe et al., 2017). For example, camels seem more tolerant to saline drinking water very high in TDS, up to 17,000 mg/L, followed by goats and then sheep (Abou Hussien et al., 1994a). Egyptian Zaraibi bucks were more tolerant than Rahmanny rams of water with a TDS concentration of 9500 mg/L, but both species were affected with a TDS concentration of 17,000 mg/L (Abou Hussien et al., 1994a, 1994b). In Hereford heifers, drinking water containing more than 5000 mg/L of TDS decreased body weight gain and feed intake during the first 51 days of the experiment though these effects were diminished later, but consumption of water with a TDS concentration of 11,000 mg/L reduced body weight gain by 49% (Saul and Flinn, 1985). Feed intake by Nguni goats was reduced by saline drinking water containing sodium chloride (NaCl) even at a low level of 5500 mg/L (Mdletshe et al., 2017). Such differences could involve salt stress reduction mechanisms that vary among species, which include a simple decrease in water consumption or an increased rate of sodium filtration (Abou Hussien et al., 1994b). Therefore, studies to understand differences among livestock species and in mechanisms of tolerance to saline drinking water will be important to develop practical recommendations for usage of these resources.

Although many of the aforementioned comments regarding research with sources of saline water focused primarily on the TDS level, specific minerals present and their levels may be important. Stanton et al. (2017) classified brackish water into four groups according to the U.S. Geological Survey (USGS) based on concentrations of specific anions, cations, and silica. Moreover, Ribeiro et al. (2021) noted that some brackish water sources in Oklahoma, USA did not fit well in any of these water types. Relatedly, apart from levels of sodium and chloride in water high in NaCl, constituents such as sulfates, carbonates, nitrates, calcium, and magnesium may have impact on physiological conditions in ruminant livestock (Mittal and Ghosh, 1983; Wagner and Engle, 2021). For example, sulfates were relatively high in brackish water used in the studies of Tsukahara et al. (2016) and Yirga et al. (2018), and sulfates can reduce feed intake, growth performance, and health (Patterson et al., 2004; Wagner and Engle, 2021).

Though much research has been conducted with ruminant livestock consuming brackish/saline water resources, there have been few comparisons of species as well as breeds. We hypothesized that sheep and goat breeds may exhibit different levels of tolerance or resilience to drinking brackish water varying in concentrations of not only TDS but also of specific minerals. Therefore, objectives of this experiment were to determine effects on intake, digestion, heat energy, ruminal fluid conditions, and blood characteristics of different breeds of young goats and hair sheep of consumption of water sources varying in TDS of a natural brackish water source and NaCl.

## 2 Materials and methods

### 2.1 Animal ethics statement

The experimental protocol was approved by the Langston University Animal Care and Use Committee (approval number: 19-001; 4 February 2019) and complied with the ARRIVE (Animal Research: Reporting of In Vivo Experiments) guidelines for animal experiments.

### 2.2 Animals, housing and feeding

Six females approximately 10 months of age at the beginning of the study of 6 animal types (AT) were used. Animal types were Angora (ANG), Boer (BOE), and Spanish (SPA) goat doelings and Dorper (DOR), Katahdin (KAT), and St. Croix (STC) ewe lambs, with initial body weight (BW) of 15.1 ± 1.30 (mean ± standard error), 17.8 ± 0.90, 15.8 ± 0.08, 34.5 ± 0.27, 31.6 ± 1.80, and 25.8 ± 0.23 kg and age of 9.8 ± 0.03, 9.1 ± 0.16, 10.2 ± 0.07, 10.0 ± 0.07, 9.6 ± 0.06, and 9.6 ± 0.08 months, respectively. The facility had a central heating and cooling system maintaining fairly constant conditions in the thermoneutral zone. Coarsely ground wheat hay was offered at approximately 120% of consumption on the preceding few days at 07:00. A mineral-vitamin supplement mixed with soybean meal (5 g/day) was top-dressed on wheat hay. In addition, a small salt (i.e., NaCl) block without other minerals was placed in the bottom of the feeders to meet the sodium requirements of all animals and to simulate a common farm management practice, although consumption was not monitored. The nutrient composition of wheat hay and supplement is presented in Table 1. Two Temperature/Relative Humidity data loggers (model number U12-011; Onset Computer Corp., Bourne, MA) were placed in different areas of the facility, with readings every 30 min. Temperature, humidity, and temperature-humidity index in each period are given in Table 2. Animals were maintained individually in 1.22 m × 1.22 m elevated pens with rubber-coated expanded metal flooring on most days of the first 2 wk of each period and were moved to metabolism crates in an adjacent room in the third week. During the first 2 wk, animals also were adapted to housing in metabolism crates fitted with “training” headboxes similar to those used with the calorimetry system.

**Table 1:** Composition of the diet offered to yearling goat doelings and hair sheep ewe lambs during 6 periods.

Item	Feedstuff	
	Concentrate	Wheat hay
Ingredient composition, % dry matter		
Vitamin premix^1^	9.904	
Trace mineral mix^2^	10.564	
Soybean meal	79.532	
Chemical composition (dry matter basis)^3^		
Ash, %	24.1 ± 0.39	9.0 ± 0.20
Crude protein, %	35.7 ± 0.34	10.0 ± 0.29
Neutral detergent fiber, %	11.9 ± 0.25	66.8 ± 0.59
Acid detergent fiber, %	10.0 ± 0.21	41.5 ± 0.58
Acid detergent lignin, %	3.0 ± 0.09	8.5 ± 0.32
Calcium, %	4.32 ± 0.113	0.37 ± 0.014
Phosphorus, %	0.53 ± 0.005	0.35 ± 0.012
Magnesium, %	0.22 ± 0.001	0.15 ± 0.006
Potassium, %	1.72 ± 0.022	1.17 ± 0.041
Sodium, %	0.069 ± 0.0040	0.066 ± 0.0100
Sulfur, %	1.78 ± 0.041	0.09 ± 0.003
Iron, mg/kg	6379 ± 261.3	201 ± 8.6
Copper, mg/kg	1799 ± 62.1	7.0 ± 2.80
Manganese, mg/kg	3474 ± 208.7	65 ± 1.7
Zinc, mg/kg	9301 ± 345.0	49 ± 12.6

1 Vitamin premix contained the following: 8,800,000 IU/kg vitamin A, 1,760,000 IU/kg vitamin D_3_, and 1100 IU/kg vitamin E.

2 Trace mineral mix contained the following: 275 mg/kg Co, 2000 mg/kg I, 43,746 mg/kg Fe, 750 mg/kg Se, 18,748 mg/kg Cu, 68,741 mg/kg Zn, and 19,998 mg/kg Mn.

3 Mean ± standard error of the mean (SEM) based on 18 weekly samples.

**Table 2:** Average daily temperature (T), relative humidity (RH), and temperature-humidity index (THI) in the facility in which animals were housed.^1^

Period	Item	Mean	SEM	Minimum	Maximum
1	T, ºC	15.1	0.04	10.6	19.8
	RH, %	42.4	0.35	23.0	91.8
	THI	58.8	0.05	52.3	64.0
2	T, ºC	16.3	0.06	11.3	21.0
	RH, %	43.0	0.35	21.6	81.3
	THI	60.2	0.07	54.4	66.3
3	T, ºC	19.3	0.07	13.6	25.8
	RH, %	56.3	0.35	30.3	88.2
	THI	64.6	0.10	56.9	73.9
4	T, ºC	20.8	0.06	14.8	26.1
	RH, %	68.5	0.28	39.1	90.5
	THI	67.3	0.09	58.4	75.9
5	T, ºC	21.6	0.03	18.8	24.8
	RH, %	70.2	0.23	44.8	89.3
	THI	68.7	0.04	64.4	74.5
6	T, ºC	24.7	0.04	20.8	30.6
	RH, %	72.7	0.21	56.6	99.1
	THI	73.7	0.06	67.3	81.9

SEM = standard error of the mean.

THI = (0.8 × T) + {RH × [(T – 14.3)/100]} + 46.3, as per Amundson et al. (2006).

1 Measures were averages of readings in two locations every 30 min.

### 2.3 Experimental design and treatments

The experiment consisted of 6 (AT or breeds) simultaneous 6 × 6 Latin squares (i.e., 6 water treatments [WT] and 6 periods), with one square for each AT. Each experimental period was 3 wk in length. Period 1 was preceded by 1 wk for adjustment to the housing conditions described earlier and then 1 wk for adaptation to the initial WT. The treatment arrangement was a 6 × 6 factorial, with 6 AT (6 animals for each AT or breed; total 36 animals) and 6 WT in each of the 6 periods. The 6 WT within each square were as follows: (1) fresh or tap water (FR), (2) the brackish water source (100-BR) used by Tsukahara et al. (2016) and Yirga et al. (2018), (3) a similar TDS level as 100-BR achieved by addition of NaCl to fresh water (100-SL), (4) BR with concentrations of all minerals present increased by approximately 50% (150-BR), (5) a similar TDS level as 150-BR achieved by addition of NaCl to fresh water (150-SL), and (6) a similar TDS level as 150-BR and 150-SL achieved by addition to 100-BR of a 1:1 mixture of BR minerals and NaCl (150-BR/SL). The NaCl added to water for SL treatments was that of Diamond Crystal Solar Naturals Salt Crystals (Cargill, Minneapolis, MN, USA), and minerals of BR added for 150-BR and 150-BR/SL were derived by evaporation of water from BR with a forced-air oven at 55 °C. Batches of all WT including FR were prepared weekly and stored in large covered plastic containers. Measured amounts of water were offered individually in molded plastic buckets for 20 min twice daily at 07:00 and 14:30 and water remaining the next morning was weighed. There was one bucket per WT in the facility to correct for evaporation loss. There were 1 to 3 days for adaptation at the beginning of periods 2 to 6 when mixtures of water of different WT were offered depending on the magnitude of change in TDS (e.g., 3 days when changed from FR to 150-BR, 150-SL, or 150-BR/SL).

### 2.4 Measures and sampling

Wheat hay offered and refusals removed daily were weighed at 07:00. Feed and water were sampled daily, and composite samples were formed for each week. Animals were weighed in the morning before feeding every 3 wk and at the beginning and end of calorimetry periods.

Ruminal fluid was sampled using a stomach tube 4 h after the morning feeding, 2 to 3 days before the calorimetry measurement phase. Ruminal fluid pH was measured with a digital meter immediately, and a 3-mL sample was placed in a tube with 2 mL of 3 mol/L HCl for ammonia analysis by the procedure of Broderick and Kang (1980). An aliquot of 4 mL was dispensed into a tube with 1 mL of 25% (wt/vol) metaphosphoric acid, stored at −20 °C and centrifuged at 2000 × *g* for 20 min before volatile fatty acid (VFA) analysis (Cottyn and Boucque, 1968) by gas chromatography (Agilent Technologies 6890N, Santa Clara, CA, USA) equipped with a capillary column (Agilent J&W DB-FFAP; 30 m length, 320 μm inner diameter and 0.25 μm film thickness) and a flame ionization detector was used under the following conditions: injection volume of 1 μL; injector temperature of 250 °C; flow rates of hydrogen, air, and nitrogen at 40, 450, and 30 mL/min, respectively; initial oven temperature of 100 °C held for 5 min and raised at 10 °C/min to 125 °C held for 5 min, and detector temperature of 250 °C.

On day 12 of each experimental period at 4 h-post feeding (approximately 3 to 4 h after drinking water was offered), blood samples were collected by jugular venipuncture into tubes containing sodium fluoride and potassium oxalate to harvest plasma and without an anticoagulant for serum. Day 12 was selected because of difficulty in sampling on days when animals were in metabolism crates. Likewise, it was felt that the likelihood of WT effects would be relatively high at this time after water was consumed rather than later. Immediately after sampling, packed cell volume (PCV) was determined with heparinized tubes (Clay Adams, Parsippany, NJ, USA) and a OSM-3 hemoximeter (Radiometer America, Westlake, OH, USA) was used to determine the concentration and oxygen saturation of hemoglobin. Blood oxygen concentration was determined as described by Eisemann and Nienaber (1990). Plasma was harvested by centrifugation for 20 min at 3000 × *g* and osmolality was measured with an osmometer (model 2020, Advanced Instruments, Inc., Norwood, MA, USA) and stored at −20 °C for further analysis. Plasma frozen at −20 °C was thawed and analyzed for glucose and lactate with a YSI 2300 STAT Plus Glucose and Lactate Analyzer (YSI Inc., Yellow Springs, OH, USA). Serum was also harvested after centrifuging for 20 min at 3000 × *g* and frozen at −20 °C, and samples were later analyzed for albumin, alkaline phosphatase, aspartate transferase, urea nitrogen, calcium, cholesterol, creatine kinase, chloride, creatinine, gamma glutamyl transferase, potassium, magnesium, sodium, and triglycerides with a Vet Axcel Chemistry Analyzer (Alfa Wassermann Diagnostic Technologies, West Caldwell, NJ, USA) according to the manufacturer's instructions. Total antioxidant activity was determined by ferric reducing activity with a kit of Arbor Assays (catalog number K043-HI; Ann Arbor, MI, USA). Aldosterone concentration was assessed with an ELISA kit (Enzo Life Sciences, Farmingdale, NY, USA).

Animals were placed in metabolism crates (0.7 m × 1.2 m; plastic-coated expanded metal floor) in the last week of each period, and feces and urine were collected on the final 4 to 5 days. Urine was acidified with 20 mL of 20% (vol/vol) sulfuric acid added in collection vessels to maintain pH below 3.0. Composite samples of feces and urine were formed by collecting 10% daily aliquots. Feed samples were analyzed for dry matter (DM), ash, crude protein (CP), acid detergent fiber, neutral detergent fiber (NDF), acid detergent lignin, and minerals at Custom Laboratory (Monett, MO, USA; http://www.customaglabs.com). Feces were analyzed for DM, ash (AOAC, 2006), nitrogen using the Doumas method in a C/N analyzer (LecoTruMac CN, St. Joseph, MI, USA), and NDF with use of heat stable amylase (Van Soest et al., 1991) and containing residual ash. Digestible energy (DE) intake was estimated assuming 19.33 kJ/g digestible organic matter (OM) intake (Garrett et al., 1959). Urine samples were analyzed for DM (lyophilization), CP (LecoTruMac CN, St. Joseph, MI, USA), and gross energy (GE) using a bomb calorimeter (Parr 6300; Parr Instrument Co., Inc., Moline, IL). Water samples were analyzed for TDS and other constituents at the Oklahoma State University Soil, Water and Forage Analytical Laboratory (SWFAL; Stillwater, OK, USA). In addition, the concentration of silica was analyzed at the American Institute for Goat Research by the ASTM (ASTM, 2000) procedure D 859-00.

The 36 animals in the third week of each period were allocated to 6 groups, with one animal per AT assigned to the different groups. Groups were cycled into metabolism crates fitted with headboxes in a calorimetry room for 1 day to measure heat energy using an indirect, open-circuit respiration calorimetry system (Sable Systems International, North Las Vegas, NV, USA). Procedures were similar to those in previous studies (Puchala et al., 2007, 2009). Oxygen concentration was assessed using a fuel cell FC-1B O_2_ analyzer (Sable Systems International, North Las Vegas, NV, USA), and CH_4_ and CO_2_ concentrations were measured with infrared analyzers (CA-1B for CO_2_ and MA-1 for CH_4_; Sable Systems International, North Las Vegas, NV, USA). Prior to gas exchange measurements, analyzers were calibrated with gases of known concentrations. An ethanol combustion test was performed to ensure complete recovery of O_2_ and CO_2_ produced with the same flow rates as used during measurements. Heat energy was calculated according to the Brouwer equation (Brouwer, 1965) with energy content of CH_4_ as 39.54 kJ/L. Urine energy expressed relative to DE intake when feces and urine were collected was multiplied by DE intake during calorimetry measures to determine urine energy loss during that time as well as intake of metabolizable energy (ME) and recovered energy (i.e., difference between ME intake and heat energy).

### 2.5 Statistical analyses

Data were analyzed with a mixed effects model with Statistical Analysis System software (Littell et al., 1998; SAS, 2011). Fixed effects in the model were AT, WT, AT × WT, and period as a repeated measure with the following model:

*Y*_*ijkl*_ = *μ* + *a*_*i*_(*AT*_*k*_) + *WT*_*j*_ + *AT*_*k*_ + *WT*_*j*_ × *AT*_*k*_ + *P*_*l*_ + *e*_*ijkl*_,

where *Y*_*ijkl*_ was the dependent variable; *μ* was the overall mean; *a*_*i*_(*AT*_*k*_) was the random effect of animal (*i* = 1–6) nested within AT (*k* = 1–6); *WT*_*j*_ was the fixed effect of water source (*j* = 1–6); *AT*_*k*_ was the fixed effect of AT (*k* = 1–6); *WT*_*j*_ × *AT*_*k*_ was the interaction between WT and AT; *P*_*l*_ was the fixed effect of period (*l* = 1–6); and *e*_*ijkl*_ was the residual error. Period was included as a repeated measure and animal within AT was the subject of the repeated measure.

When the AT × WT was significant (*P* < 0.05), the analysis was conducted by AT. Differences among means were determined by least significant difference with a protected F-test (*P* < 0.05). There were very few significant effects of WT or interactions with AT, although numerous differences among AT existed. Therefore, *P*-values and AT means are presented in the main tables of the article and the majority of WT main effect means and those for the AT × WT interaction appear in supplemental tables (Tables S1–S5) so that they could potentially be used in future meta-analysis studies.

## 3 Results

### 3.1 Water composition

As expected, concentrations of TDS and all minerals were lower for FR than for other WT (Table 3). Concentrations of sodium and chloride were greater and those of other constituents such as sulfate, calcium, magnesium, and silica were lower for 100-SL and 150-SL than for 100-BR and 150-BR. The TDS levels in 100-SL and 150-SL that were expected to be similar to 100-BR and 150-BR, respectively, were greater than anticipated. The TDS level for 150-BR relative to 100-BR was slightly higher, whereas those for 150-SL and 150-BR/SL relative to 100-BR and 100-SL, respectively, were slightly lower than expected.

**Table 3:** Composition of water consumed by yearling goat doelings and hair sheep ewe lambs of different breeds.^1^

Item	FR		100-BR		100-SL		150-BR		150-SL		150-BR/SL	
	Mean	SEM	Mean	SEM	Mean	SEM	Mean	SEM	Mean	SEM	Mean	SEM
pH	8.36	0.077	7.97	0.035	8.15	0.073	8.09	0.021	8.06	0.086	8.00	0.045
Electrical conductivity, μS/cm	313	10.2	7249	115.5	8612	476.4	10,597	178.9	12,589	536.0	11,060	716.4
Total dissolved solids, mg/kg	209	7.1	4928	83.4	5684	315.7	7508	123.5	8309	353.8	7319	472.2
Hardness, mg/kg	114	2.2	810	45.7	135	57.9	1169	30.7	26	5.4	808	51.3
Calcium, mg/kg	17.3	0.59	224.9	13.22	31.7	17.01	326.9	8.79	7.2	1.72	236.3	16.71
Chloride, mg/kg	20	2.2	1175	45.3	2472	142.6	1778	75.6	3717	192.0	2411	167.6
Magnesium, mg/kg	17.1	0.30	60.5	3.18	15.7	4.62	86.0	3.62	2.6	0.68	53.1	2.75
Potassium, mg/kg	6.25	1.019	4.59	0.285	4.94	0.585	6.53	0.948	3.81	0.702	3.88	0.831
Sodium, mg/kg	15	1.5	1387	24.0	1768	106.9	2168	38.6	2698	144.1	2290	154.0
Sulfate, mg/kg	7	0.2	1962	123.6	191	168.9	3052	81.4	30	2.5	1938	124.3
Boron, mg/kg	0.08	0.003	8.3	0.54	0.8	0.72	12.8	0.46	0.0	0.01	8.3	0.56
Silica, mg/L	1.01	0.095	4.47	0.167	0.94	0.108	6.40	0.160	0.94	0.116	4.36	0.137

SEM = standard error of the mean.

1 FR = fresh water; 100-BR = brackish water (BR); 100-SL = NaCl added to FR for a similar total dissolved solids (TDS) level as 100-BR; 150-BR = 100-BR plus addition of approximately 50% of 100-BR TDS; 150-SL = NaCl added to FR for a similar TDS level as 150-BR; 150-BR/SL = a similar TDS level as 150-BR and 150-SL by addition to 100-BR of a 1:1 mixture of BR minerals and NaCl; 18 weekly composite samples.

### 3.2 Intake, digestion, and calorimetry measures

The BW of animals differed (*P* < 0.01) among AT and, naturally, average DM and water intake in grams per day during the entire experiment varied (*P* < 0.05) considerably with AT (Table 4). Among AT, DM intake as a percentage of BW was lowest for ANG and higher for STC than for BOE and SPA (*P* < 0.05), but was similar for DOR, KAT, BOE, and SPA. Water intake in a percentage of BW was lower for SPA vs. ANG (*P* < 0.05) and intermediate for BOE (*P* > 0.05), but was similar among sheep breeds (*P* > 0.05). Water intake relative to DM intake was higher for ANG than for other AT (*P* < 0.05).

**Table 4:** Effects of water treatment and breed of yearling goat doelings and hair sheep ewe lambs on body weight, water and feed intake, digestion, and energy metabolism.

Item	*P*-values^1^			AT^2^						SEM
	WT	AT	WT × AT^3^	ANG	BOE	SPA	DOR	KAT	STC	
Entire 3-wk periods										
BW, kg	0.99	<0.01	0.99	17.0^a^	20.4^a^	17.5^a^	38.1^b^	34.3^b^	30.1^b^	1.28
DM intake										
g/day	0.86	<0.01	1.00	351^a^	520^b^	454^ab^	1045^c^	1020^c^	930^c^	50.0
% BW	0.57	<0.01	0.92	2.04^a^	2.54^b^	2.58^b^	2.72^bc^	2.99^bc^	3.06^c^	0.161
Water intake										
g/day	0.04	<0.01	0.69	871^a^	828^a^	682^a^	1775^bc^	1944^c^	1482^b^	109.9
% BW	0.04	0.02	0.51	5.21^bc^	4.06^ab^	3.88^a^	4.65^abc^	5.69^c^	4.97^abc^	0.388
g/g DM intake	0.15	<0.01	0.73	2.81^b^	1.60^a^	1.53^a^	1.74^a^	1.97^a^	1.65^a^	0.159
Digestion measure days										
Dry matter										
Intake, g/day	0.98	<0.01	0.97	370^a^	556^b^	497^ab^	1066^cd^	1094^d^	950^c^	49.0
Digestion, %	0.22	0.04	0.19	59.2^a^	62.5^abc^	62.9^abc^	64.1^bc^	66.2^c^	62.1^ab^	1.38
Digested, g/day	0.86	<0.01	0.97	225^a^	348^b^	314^ab^	688^cd^	725^d^	597^c^	34.8
Organic matter										
Intake, g/day	0.98	<0.01	0.97	336^a^	505^b^	452^ab^	970^cd^	995^d^	864^c^	44.6
Digestion, %	0.25	0.02	0.18	62.0^a^	65.4^ab^	65.2^ab^	67.1^bc^	69.1^c^	65.3^ab^	1.29
Digested, g/day	0.89	<0.01	0.97	213^a^	331^b^	297^ab^	655^cd^	689^d^	570^c^	32.6
NDF										
Intake, g/day	0.98	<0.01	0.97	244^a^	369^b^	329^ab^	709^cd^	728^d^	631^c^	32.7
Digestion, %	0.37	0.02	0.12	62.6^a^	67.4^b^	66.7^b^	68.3^b^	70.3^b^	66.9^b^	1.35
Digested, g/day	0.94	<0.01	0.98	156^a^	248^b^	221^ab^	487^cd^	513^d^	427^c^	23.7
Nitrogen										
Intake, g/day	0.98	<0.01	0.93	5.9^a^	9.0^b^	8.1^ab^	17.2^cd^	17.6^d^	15.3^c^	0.80
Digestion, %	0.16	0.20	0.12	61.6	63.2	63.7	63.3	65.7	60.3	1.51
Digested, g/day	0.76	<0.01	0.90	3.8^a^	5.7^b^	5.3^ab^	10.9^cd^	11.6^d^	9.3^c^	0.58
Calorimetry measure days										
Dry matter intake, g/day	0.62	<0.01	0.86	299^a^	495^b^	394^ab^	999^d^	1009^d^	834^c^	50.2
GE intake, MJ/day	0.62	<0.01	0.86	5.24^a^	8.69^b^	6.93^ab^	17.57^d^	17.73^d^	14.66^c^	0.884
DE intake, MJ/day	0.89	<0.01	0.89	3.33^a^	5.69^b^	4.55^ab^	11.85^d^	12.30^d^	9.66^c^	0.618
Heart rate, beats/min	0.01	0.42	0.17	75.5	84.7	85.0	79.6	79.7	79.8	3.46
Respiratory quotient	0.47	<0.01	0.31	0.883^a^	0.952^b^	0.930^b^	0.986^c^	0.997^c^	0.990^c^	0.0113
Heat energy, MJ/day	0.41	<0.01	0.91	2.80^a^	3.77^b^	3.28^ab^	7.06^d^	6.53^cd^	5.85^c^	0.261
Heat energy, kJ/kg BW^0.75^	0.28	<0.01	0.56	349^a^	397^b^	388^ab^	453^c^	459^c^	440^c^	14.2
Heat energy, % DE intake	0.75	<0.01	0.47	105^c^	79^ab^	83^bc^	64^ab^	59^a^	68^ab^	7.9
Methane energy, MJ/day	0.52	<0.01	0.77	0.32^a^	0.62^b^	0.47^ab^	1.26^c^	1.21^c^	1.09^c^	0.062
Methane energy, % GE intake	0.81	0.53	0.52	6.61	7.27	7.24	7.44	7.31	7.73	0.429
Methane energy, % DE intake	0.83	0.62	0.78	10.89	12.78	11.27	11.13	10.68	12.17	0.973
Urine energy, MJ/day	0.58	<0.01	0.95	0.09^a^	0.15^bc^	0.10^ab^	0.26^d^	0.24^d^	0.18^c^	0.016
ME intake, MJ/day	0.91	<0.01	0.86	2.91^a^	4.93^b^	3.98^b^	10.34^d^	10.85^d^	8.39^c^	0.554
Recovered energy, MJ/day	0.92	<0.01	0.66	0.11^a^	1.16^a^	0.69^a^	3.28^bc^	4.32^c^	2.54^b^	0.431

SEM = pooled standard error of the mean for animal type effect; BW = body weight; DM = dry matter; NDF = neutral detergent fiber; GE = gross energy; DE = digestible energy; ME = metabolizable energy.

^a,b,c,d^Means in a row without a common superscript letter differ (*P* < 0.05).

1 AT = animal type; WT = water treatment.

2 ANG = Angora goats; BOE = Boer goats; SPA = Spanish goats; DOR = Dorper sheep; KAT = Katahdin sheep; STC = St. Croix sheep.

3 Mean values by breed × water treatment are presented in Tables S1–S3.

Digestion coefficients for DM and OM were similar among goat breeds (*P* > 0.05) and for sheep were greater for KAT than for STC (*P* < 0.05), with an intermediate value for DOR (*P* > 0.05; Table 4). Conversely, NDF digestion was lowest among AT for ANG (*P* < 0.05), with values similar among the other five AT (*P* > 0.05). Digestibility of nitrogen was similar among all AT (*P* > 0.05).

During calorimetric measurement days, differences in DM intake (g/day) were similar to those in the entire 3-wk periods, and those in energy intake (MJ/day) and urinary energy (MJ/day) followed ones in DM intake (Table 4). Heart rate was similar among AT (*P* > 0.05). The respiratory quotient was lower for ANG than for BOE and SPA but was similar among sheep breeds, which had values greater than for goats. Heat energy as a percentage of DE intake was greater for ANG than for other breeds except SPA (*P* < 0.05). Heat energy for KAT was less than for SPA (*P* < 0.05), with similar values for BOE, DOR, KAT, and STC (*P* > 0.05). Methane emission as a percentage of DE intake did not differ among AT (*P* > 0.05). Recovered energy (MJ/day) was lower for goats than for sheep, with values similar among goat breeds. Recovered energy was greater for KAT vs. STC (*P* < 0.05), although values were similar for KAT and DOR.

Water intake in gram per day and a percentage of BW was greater for 150-SL than for other WT (*P* < 0.05) except 150-BR/SL, with values similar between 150-SL and 150-BR/SL (*P* > 0.05; Table 5). Heart rate during the days of calorimetry measures was greatest among WT for 150-BR/SL (*P* < 0.05).

**Table 5:** Effects of water treatment on water intake and heart rate by yearling goat doelings and hair sheep ewe lambs of different breeds.

Item	Water treatment^1^						SEM
	FR	100-BR	100-SL	150-BR	150-SL	150-BR/SL	
Entire 3-wk periods							
Water intake							
g/day	1230^a^	1185^a^	1244^a^	1188^a^	1427^b^	1307^ab^	70.7
% BW	4.66^a^	4.50^a^	4.61^a^	4.42^a^	5.30^b^	4.96^ab^	0.249
Calorimetry measure days							
Heart rate, beats/min	78.2^a^	81.2^a^	80.6^a^	78.5^a^	79.3^a^	86.1^b^	2.07

SEM = pooled standard error of the mean for the water treatment effect; BW = body weight.

^a,b^Means within grouping without a common superscript letter differ (*P* < 0.05).

1 FR = fresh water; 100-BR = brackish water (BR); 100-SL = NaCl added to FR for a similar total dissolved solids (TDS) level as 100-BR; 150-BR = 100-BR plus addition of approximately 50% of 100-BR TDS; 150-SL = NaCl added to FR for a similar TDS level as 150-BR; 150-BR/SL = a similar TDS level as 150-BR and 150-SL by addition to 100-BR of a 1:1 mixture of BR minerals and NaCl.

### 3.3 Ruminal fluid and blood measures

Ruminal pH was lower for goat than for sheep breeds (*P* < 0.05; Table 6). Ruminal ammonia nitrogen concentration was highest among AT for BOE (*P* < 0.05) and higher for SPA than for ANG, KAT, and STC (*P* < 0.05). In accordance with ruminal pH, the total VFA concentration was greater or tended to be greater for goats than sheep. The molar percentage of acetate was greater for ANG and BOE than for other AT (*P* < 0.05). There were some differences in the molar percentage of propionate, but the maximum difference was only 1.3 percentage units. The molar percentage of isobutyrate was lower for goat than for sheep breeds (*P* < 0.05). There was an interaction between AT and WT in the molar percentage of butyrate (*P* = 0.04). The statistical analysis by AT revealed that the WT effect occurred only in KAT, with a lower value for 150-BR than for 100-BR, 100-SL, and 150-BR/SL (*P* < 0.05; Table 7). The molar percentage of isovalerate was lowest among AT for ANG (*P* < 0.05) and higher for DOR and KAT than for BOE and SPA (*P* < 0.05; Table 6). The molar percentage of valerate was lower for ANG and BOE than for the sheep breeds (*P* < 0.05), with the valerate percentage for SPA less than for KAT (*P* < 0.05).

**Table 6:** Effects of water treatment and breed of yearling goat doelings and hair sheep ewe lambs on ruminal fluid characteristics and blood constituent concentrations.

Item	*P*-values^1^			AT^2^						SEM
	WT	AT	WT × AT^3^	ANG	BOE	SPA	DOR	KAT	STC	
Ruminal fluid										
pH	0.58	<0.01	0.63	5.84^a^	5.85^a^	5.82^a^	6.06^b^	6.11^b^	6.11^b^	0.035
Ammonia nitrogen, mg/dL	0.61	<0.01	0.15	6.0^a^	10.0^c^	8.0^b^	6.6^ab^	5.8^a^	6.2^a^	0.54
Total VFA, mmol/L	0.85	<0.01	0.14	58.7^bc^	59.3^bc^	62.2^c^	55.0^ab^	52.3^a^	56.6^ab^	1.65
Acetate, molar %	0.10	<0.01	0.58	81.2^b^	81.3^b^	79.6^a^	79.0^a^	79.1^a^	79.0^a^	0.41
Propionate, molar %	0.18	0.01	0.95	11.6^ab^	11.5^a^	12.8^c^	12.3^bc^	12.5^c^	12.2^abc^	0.35
Acetate:propionate	0.15	<0.01	0.96	7.05^bc^	7.22^c^	6.40^a^	6.51^a^	6.40^a^	6.56^ab^	0.170
Isobutyrate, molar %	0.72	<0.01	0.85	0.22^a^	0.30^b^	0.32^b^	0.46^c^	0.44^c^	0.40^c^	0.026
Butyrate, molar %	0.31	<0.01	0.04	6.38^ab^	6.18^a^	6.50^ab^	7.23^cd^	6.90^bc^	7.56^d^	0.218
ANG	0.11									
BOE	0.78									
SPA	0.33									
DOR	0.53									
KAT	0.04									
STC	0.33									
Isovalerate, molar %	0.62	<0.01	0.73	0.15^a^	0.27^b^	0.29^bc^	0.47^d^	0.47^d^	0.39^cd^	0.036
Valerate, molar %	0.14	<0.01	0.59	0.42^a^	0.42^a^	0.47^ab^	0.50^bc^	0.53^c^	0.51^bc^	0.019
Blood measures										
PCV, %	0.08	<0.01	0.92	21.4^b^	18.1^a^	23.5^b^	31.1^c^	29.6^c^	29.8^c^	1.06
tHb, g/dL	0.04	<0.01	0.77	8.6^b^	7.0^a^	9.5^b^	12.1^c^	11.8^c^	11.5^c^	0.43
Hb O_2_ saturation, %	0.94	<0.01	0.69	74.3^c^	69.9^bc^	67.0^ab^	61.3^a^	67.0^ab^	69.4^b^	2.10
O_2_ concentration, mmol/L	0.45	<0.01	0.74	8.8^b^	6.7^a^	8.7^b^	10.3^c^	10.9^c^	11.1^c^	0.47
Glucose, mg/dL	0.23	<0.01	0.69	43.7^a^	51.2^b^	52.7^b^	57.1^c^	52.8^b^	56.2^c^	1.16
Lactate, mg/dL	0.13	<0.01	0.41	10.7^a^	12.9^a^	16.7^a^	31.3^b^	17.6^a^	18.2^a^	2.92
Albumin, g/dL	0.15	<0.01	1.00	2.35^b^	2.02^a^	2.35^b^	2.46^b^	2.31^b^	2.39^b^	0.068
Urea nitrogen, mg/dL	0.53	<0.01	0.98	15.4^b^	18.0^c^	13.9^ab^	11.3^ab^	10.6^a^	11.7^ab^	1.38
Triglycerides, mg/dL	0.24	0.24	0.32	28.6	27.3	28.1	30.7	25.5	25.5	1.62
Cholesterol, mg/dL	0.94	<0.01	0.83	74.8^b^	51.3^a^	51.0^a^	58.4^a^	56.2^a^	59.4^a^	3.39
Creatinine, mg/dL	0.49	<0.01	0.67	0.72^a^	0.86^c^	0.82^bc^	1.02^d^	0.84^bc^	0.77^ab^	0.032
Alkaline phosphatase, U/L	0.95	0.02	0.98	49^a^	39^a^	298^b^	108^a^	105^a^	140^a^	52.7
Aspartate transferase, U/L	0.34	<0.01	0.14	139^c^	65^ab^	63^a^	95^b^	72^ab^	72^ab^	11.1
Gamma glutamyl transferase, U/L	0.29	<0.01	0.28	105^c^	38^a^	37^a^	63^b^	59^b^	52^ab^	5.0
Creatine kinase, U/L	<0.01	<0.01	<0.01	190^c^	153^bc^	110^ab^	82^a^	80^a^	83^a^	17.3
ANG	<0.01									
BOE	0.05									
SPA	0.77									
DOR	0.23									
KAT	0.61									
STC	0.55									
Osmolality, mOsm/kg	0.67	0.06	0.12	296	302	301	302	298	299	1.7
Calcium, mg/dL	0.71	<0.01	0.23	8.70^a^	8.59^a^	8.84^ab^	9.46^bc^	9.59^c^	9.82^c^	0.236
Chloride, mmol/L	0.81	0.13	0.57	115	114	114	113	112	113	0.8
Potassium, mmol/L	0.83	0.01	0.46	4.99^ab^	4.83^a^	4.82^a^	5.27^b^	5.24^b^	5.28^b^	0.114
Magnesium, mg/dL	0.44	0.02	0.66	2.00^a^	2.19^b^	2.23^b^	2.28^b^	2.35^b^	2.18^ab^	0.064
Sodium, mmol/L	0.94	0.17	0.29	144	144	144	145	141	141	1.1
Total antioxidant capacity, μmol/L	0.43	<0.01	0.96	241^c^	221^bc^	191^a^	192^a^	199^ab^	202^ab^	9.46
Aldosterone, pg/mL	<0.01	0.51	0.80	93	157	97	93	74	112	30.9

SEM = pooled standard error of the mean for the animal type effect; VFA = volatile fatty acids; PCV = packed cell volume; tHb = total hemoglobin; Hb O_2_ = oxygen saturation of hemoglobin.

^a,b,c,d^Means in a row without a common superscript letter differ (*P* < 0.05).

1 WT = water treatment; AT = animal type.

2 ANG = Angora goats; BOE = Boer goats; SPA = Spanish goats; DOR = Dorper sheep; KAT = Katahdin sheep; STC = St. Croix sheep.

3 Mean values by breed × water treatment are presented in Tables S4 and S5.

**Table 7:** Effects of water treatment and breed × water treatment on ruminal fluid butyrate concentration, blood concentrations of total hemoglobin, creatine kinase, and aldosterone in yearling goat doelings and hair sheep ewe lambs.

Item	Animal type^1^	Water treatment^2^						SEM
		FR	100-BR	100-SL	150-BR	150-SL	150-BR/SL	
Ruminal fluid								
Butyrate, molar %	ANG	6.03	6.63	6.23	7.49	6.61	5.31	0.532
	BOE	6.45	6.30	6.21	6.36	6.00	5.78	0.414
	SPA	6.80	6.31	6.03	7.13	6.42	6.29	0.374
	DOR	7.34	7.41	7.41	7.04	6.90	7.31	0.237
	KAT	6.89^ab^	7.07^b^	7.14^b^	6.32^a^	6.70^ab^	7.23^b^	0.309
	STC	7.61	7.88	7.36	7.28	7.81	7.40	0.287
Blood constituents								
tHb, g/dL	Mean	10.0^ab^	9.9^a^	10.2^ab^	10.5^b^	10.2^ab^	9.7^a^	0.24
Creatine kinase, U/L	ANG	358^c^	137^a^	132^a^	164^ab^	154^ab^	262^bc^	57.6
	BOE	170^b^	168^b^	151^ab^	133^a^	147^ab^	148^ab^	14.0
	SPA	112	114	116	107	113	101	10.0
	DOR	87	90	87	83	74	72	8.9
	KAT	69	73	80	81	79	97	11.1
	STC	88	82	82	90	77	82	8.2
Aldosterone, pg/mL	Mean	151^b^	104^a^	90^a^	86^a^	92^a^	102^a^	16.7

SEM = pooled standard error of the mean for the water treatment effect; tHb = total hemoglobin.

^a,b,c^Means within grouping without a common superscript letter differ (*P* < 0.05).

1 ANG = Angora goats; BOE = Boer goats; SPA = Spanish goats; DOR = Dorper sheep; KAT = Katahdin sheep; STC = St. Croix sheep.

2 FR = fresh water; 100-BR = brackish water (BR); 100-SL = NaCl added to FR for a similar total dissolved solids (TDS) level as 100-BR; 150-BR = 100-BR plus addition of approximately 50% of 100-BR TDS; 150-SL = NaCl added to FR for a similar TDS level as 150-BR; 150-BR/SL = a similar TDS level as 150-BR and 150-SL by addition to 100-BR of a 1:1 mixture of BR minerals and NaCl.

There were differences among AT in the majority of blood variables, but WT only affected hemoglobin and aldosterone concentrations, with the sole AT × WT interaction for the level of creatine kinase (Table 6). The PCV and concentration of hemoglobin ranked (*P* < 0.05) BOE < ANG and SPA < DOR, KAT, and STC. The hemoglobin concentration was lower (*P* < 0.05) for 100-BR and 150-BR/SL than for 150-BR (Table 7). Oxygen saturation of hemoglobin was lower for DOR than for other AT (*P* < 0.05) except KAT and SPA and higher for ANG than for other AT (*P* < 0.05) except BOE (Table 6). The hemoglobin concentration and oxygen saturation resulted in a blood oxygen concentration ranking (*P* < 0.05) of BOE < ANG and SPA < DOR, KAT, and SPA, and goats had a lower blood oxygen concentration than sheep (*P* < 0.05).

The glucose concentration ranked (*P* < 0.05) ANG < BOE, SPA, and KAT < DOR and STC (Table 6). The lactate concentration was considerably greater for DOR than for other AT (*P* < 0.05). The albumin concentration was lower (*P* < 0.05) for BOE than other AT that had similar concentrations. The level of urea nitrogen was highest among AT for BOE (*P* < 0.05) and greater for ANG vs. KAT (*P* < 0.05). The triglyceride concentration was similar among AT (*P* > 0.05), whereas the level of cholesterol was greater (*P* < 0.05) for ANG than for other AT that had similar concentrations. The creatinine level was highest among AT for DOR (*P* < 0.05) and higher for BOE than for STC and ANG (*P* < 0.05).

Alkaline phosphatase activity was much greater for SPA than for other AT (*P* < 0.05; Table 6). Aspartate transferase and gamma glutamyl transferase activities were highest among AT for ANG (*P* < 0.05). There was an AT × WT interaction in the concentration of creatine kinase (*P* < 0.01), with differences among WT for ANG and BOE but not for other breeds (Table 6, Table 7). The creatine kinase activity for ANG was higher for FR than for other treatments (*P* < 0.05) except 150-BR/SL (Table 7). For BOE, activities for FR and 100-BR were greater than for 150-BR (*P* < 0.05), with intermediate values for other treatments (*P* > 0.05).

Plasma osmolality was similar among AT (*P* > 0.05; Table 6). The calcium concentration was greater for KAT and STC than for all goat breeds (*P* < 0.05), with an intermediate value for DOR (*P* > 0.05). There were no AT differences in concentrations of chloride, sodium, or aldosterone. There was a difference among WT in the concentration of aldosterone, with the higher value for FR (*P* < 0.05) than other WT (Table 7). The level of potassium was higher for the three sheep breeds than for BOE and SPA (*P* < 0.05), with an intermediate value for ANG (*P* > 0.05). The concentration of magnesium was lower for ANG than for BOE, SPA, DOR, KAT (*P* < 0.05), and STC (*P* = 0.06). Total antioxidant capacity for ANG was highest among AT (*P* < 0.05) except for BOE and lower for SPA and DOR than for BOE (*P* < 0.05).

## 4 Discussion

### 4.1 Water composition and treatments

Water containing TDS higher than 1000 mg/L is usually considered unfit for human consumption, with sources between 1000 and 10,000 mg/L often termed “brackish” (https://www.usgs.gov/mission-areas/water-resources/science/national-water-census-brackish-groundwater-assessment/, accessed on 13 November 2022). Sources of brackish water in the USA have been categorized into four groups by the USGS based on salinity and levels of specific anions and cations (i.e., sodium bicarbonate, calcium sulfate, and NaCl-dominant for groups 1, 2, and 3, respectively) as well as silica content (group 4) (Stanton et al., 2017). In the present study, 100-BR had a TDS level near 5000 mg/kg, with NaCl at approximately 2500 mg/kg and a high concentration of sulfates (i.e., 1962 mg/kg), which could be characterized as ‘sodium sulfate-dominant.’ In this regard, these characteristics do not fit well into any of the USGS groups, as Ribeiro et al. (2021) also found for some other brackish water sources in Oklahoma, USA. The composition of FR and 100-BR differed slightly from sources used in previous studies of Tsukahara et al. (2016) and Yirga et al. (2018). Concentrations of TDS, sulfate, calcium, sodium, magnesium, and chloride and electrical conductivity, pH, and hardness in FR were lower, but concentrations of potassium and boron were greater in the present study than in FR used earlier (Tsukahara et al., 2016; Yirga et al., 2018). Concentrations of boron, potassium, sodium, calcium, magnesium, chloride, sulfate, and TDS and electrical conductivity, pH, alkalinity, and hardness in 100-BR were lower than in previous studies (Tsukahara et al., 2016; Yirga et al., 2018). As suggested by Yirga et al. (2018), differences in the composition of water from the same source at different times could relate to residence time in the aquifer, temperature, water table depth, recharge rate, etc. The concentrations of most constituents of the brackish water treatments of this experiment can be considered safe based on maximum levels recommended for ruminant livestock by Socha et al. (2003). However, levels of calcium and hardness (i.e., 100-BR, 150-BR and 150-BR/SL) and sodium and chloride concentrations in all WT other than the FR control were relatively high. But, the WT of Yirga et al. (2018) with a high level of NaCl added to the brackish water source entailed higher concentrations of sodium and chloride than any treatments of the current study without observing deleterious effects on the sheep and goats used. The high sulfate concentration in brackish water of this and previous studies (Tsukahara et al., 2016; Yirga et al., 2018) could cause copper, molybdenum, and thiamine deficiencies and polioencephalomalacia (Olkowski et al., 1991) in ruminants fed diets with lower concentrations of these constituents, which should be tested in a long-term experiment.

The only variables affected by WT were water intake, blood creatinine kinase concentration, and heart rate. In the study of Patra et al. (2023) with Boer, Spanish, and Tennessee Stiff-Leg does and Dorper, Katahdin, and St. Croix ewes consuming TDS levels up to 100 % greater than in the natural source, only a few variables were also significant among the treatments such water intake, plasma osmolality, total blood aldosterone levels, VFA concentration, molar proportion of butyrate, and blood phosphorus concentration. It is also plausible that availability of NaCl blocks resulted in greater block consumption for the FR treatment (Bahman et al., 1993; Runa et al., 2019) as well as differences among other WT. Though this may have contributed to a few WT effects, it was not preferable to imposing impractical farm conditions that were possibly conducive to a sodium deficiency.

### 4.2 Water intake

Water intake is governed by palatability and thirst. In a free-choice system, animals can differentiate water salinity taste and have innate regulatory mechanisms to adjust NaCl intake as per their requirement (Enke et al., 2022; Runa et al., 2019). The 20% greater water intake for 150-SL compared with 150-BR occurred despite a similar TDS level. The level of sodium in 150-SL was much greater than in 150-BR, with levels of other minerals much less in 150-SL. These findings suggest that the sodium concentration is an important factor in stimulating the thirst center in the brain, presumably because of an increased concentration of sodium in blood (Stachenfeld, 2008). This may be required to maintain the extracellular sodium concentration and plasma tonicity if the kidneys fail to optimally concentrate urine to adequately excrete the sodium load (Kii and Dryden, 2005). It has been suggested that the thirst center is very sensitive to elevated sodium concentration and osmolality in plasma with only a 2% to 3% increase stimulating a sense of thirst (Stachenfeld, 2008). In this context, in a study with either NaCl (7000 mg/L; 8045 mg/L of TDS and 2460 mg/L of sodium) or sodium sulfate (2000 mg/L; 2645 mg/L of TDS and 1230 mg/L of sulfate) added to tap water (760 mg/L TDS, 70 mg/L sodium, and 105 mg/L sulfate), water intake was greatest for the NaCl WT (Yousfi et al., 2016), suggesting a considerable impact of sodium. However, as noted later, the sodium concentration in jugular venous blood was not influenced by WT.

In many studies, water intake by different ruminant species has risen as the water salinity level via NaCl addition increased. These include greater intake than of tap water for Barbarine lambs consuming water with 8000 mg/L TDS (Yousfi et al., 2016), Barbarine rams with 12,400 and 16,000 mg/L (Yousfi and Salem, 2017), deer with 1000 to 8500 mg/kg (Kii and Dryden, 2005), lactating Alpine goats and Santa Inês lambs with 3200 to 8330 mg/L (Costa et al., 2021; de Albuquerque et al., 2020), and Red Sindhi heifers with 3200 to 8330 mg/L (Alves et al., 2017). In slight contrast, with growing and mature Boer goat wethers and mature Katahdin sheep wethers, the mature Boer goat was the only animal type for which water intake increased when the TDS level was elevated by an increasing level of this brackish water source of the present experiment (Yirga et al., 2018).

There are also some studies in which very high salinity and TDS concentrations reduced water intake. For instance, in grazing Merino sheep, water intake was greater with TDS concentrations between 12,000 and 16,000 mg/L achieved by adding NaCl and magnesium sulfate relative to fresh water, but intake was reduced when the TDS level was raised to 20,000 mg/L (Wilson, 1975). Similarly, increasing salinity by addition of NaCl (500, 5000, 10,000, and 20,000 mg/L) increased water intake by adult male goats until a level of 10,000 mg/L from 2.0 to 3.2 L/day, but intake was decreased to 2.5 L/day with NaCl at 20,000 mg/L (Zoidis and Hadjigeorgiou, 2017). However, decreases in water intake have also been observed with lower levels of TDS. For example, consumption of water with a TDS level of 7478 mg/L via addition of NaCl and sodium sulfate by Braford × Criollo Argentino calves was less than of tap water (López et al., 2016). With Murrah buffalo calves, intake of water with TDS concentrations of 8790 mg/L and 6110 mg/L achieved by dilution with tap water (560 mg/L of TDS) was less than of the tap water alone (Sharma et al., 2017). In female adult Nguni goats, water intake decreased when the TDS level was elevated mainly by adding NaCl at 5500 and 11,000 mg/L (Mdletshe et al., 2017). In fact, tolerable levels of NaCl and TDS will vary with many factors, including species, breed, physiological stage of animals, diet composition, history of exposure, and environmental conditions (López et al., 2021). Because of the relationship between DM and water intake (Winchester and Morris, 1956), greater water intake (g/day) by sheep than goats in the present experiment also may relate to the species difference in DM intake (g/day).

### 4.3 Feed intake and digestion

Greater feed intake for sheep than for goats is due in large part to differences in BW. Also, feed intake relative to BW varied among breeds and was greater for sheep than for goats, which might have contributed to greater heat energy production and recovered energy for sheep in the present study. Likewise, Herselman et al. (1999) reported that ME intake was similar in both sheep and goats, but heat energy was greater in sheep than goats grazing grass-based pasture. The numerically greatest water intake for % BW among goat breeds for ANG may have contributed to lowest NDF digestibility as a result of increased ruminal digesta passage rate and reduced retention time (Attia-Ismail et al., 2008; McGregor, 2004), despite considerably lower DM intake for % BW for ANG than for BOE and SPA, resulting in a higher ratio of water to DM intake.

Consumption of saline water also has had no negative effects on feed intake or digestibility in many other studies (Attia-Ismail et al., 2008; de Araújo et al., 2019; Moehlenpah et al., 2021; Tsukahara et al., 2016; Yirga et al., 2018). Levels of TDS in water up to 8300 mg/L with Santa Inês sheep (de Albuquerque et al., 2020; Moura et al., 2016), 6900 mg/L with Spanish and Boer goats (Tsukahara et al., 2016), and 8300 mg/L with Red Sindi heifers (Alves et al., 2017) did not affect feed intake. But in other studies, saline drinking water has influenced feed intake. For example, feed intake was reduced by consumption of water with NaCl added for TDS of 12,400 and 16,000 mg/L with Barbarine rams (Yousfi and Salem, 2017), 9500 mg/L with Rahmany rams, and 17,000 mg/L with both Rahmany rams and Zaraibi bucks (Abou Hussien et al., 1994a). With Murrah buffalo calves, DM intake was lower when well water with 8790 mg/L TDS was consumed relative to water with lower TDS of 2570, 4470, and 6110 mg/L through dilution with tap water (Sharma et al., 2017). Exact mechanisms by which high levels of minerals in drinking water reduce feed intake are unclear and most likely variable. For example, hormones (e.g., vasopressin) and neuronal pathways including chemo- and mechanoreceptors in the rumen that can have regulatory roles in feed intake control have been impacted by high NaCl intake in some instances (Blache et al., 2007). In accordance, slower ruminal particulate passage rate, longer ruminal digesta retention time, and greater digesta and fluid volume/fill in the reticulorumen have been observed with consumption of water high in TDS, which may contribute to relatively low feed intake (Kattnig et al., 1992).

In another experiment conducted with this source of brackish water (Moehlenpah et al., 2021), beef cows and growing beef heifers received water treatments of fresh, 50% fresh:50% brackish, 100% brackish (5263 mg/L of TDS), and fresh water with NaCl added for similar TDS levels. Somewhat similar to results of the current experiment, water treatments had no or minor effects on water and feed intake and digestibility (Moehlenpah et al., 2021). Likewise, feed intake, nutrient digestion, and nitrogen balance in deer were not affected by TDS up to 8500 mg/kg by addition of NaCl (Kii and Dryden, 2005). In Murrah buffalo calves, consumption of water with up to 8790 mg/L TDS did not influence digestibility of DM, CP, or fiber components or urinary nitrogen excretion, but nitrogen retention was lower at the highest level of TDS because of decreased feed intake (Sharma et al., 2017). Similarly, in Santa Inês sheep a water TDS concentration up to 8320 mg/L via addition of NaCl did not affect digestibility or nitrogen balance (de Albuquerque et al., 2020).

Contrary to the studies cited above, in some cases water relatively high in TDS has decreased digestibility of nutrients (Abou Hussien et al., 1994a; Yirga et al., 2018). In the study of Abou Hussien et al. (1994a), a TDS level of 16,900 mg/L achieved by addition of NaCl to tap water decreased digestibility of DM, CP, and fiber components in both Rahmany rams and Zaraibi bucks compared with tap water (256 mg/L of TDS), but water with TDS of 9470 mg/L did not have impact. In growing and mature Boer goat wethers and mature Katahdin sheep wethers subjected to water treatments of 100% fresh, 50% fresh:50% brackish, 100% brackish (5596 mg/L TDS), and 100% brackish plus 3450 g/L or 6900 g/L of added NaCl, digestibilities of OM, CP, and NDF were lower for treatments containing brackish water compared with fresh water in growing Boer goats only (Yirga et al., 2018). From the above discussions, it is understandable that digestion is influenced by TDS concentration (Abou Hussien et al., 1994a), physiological stage, and breed (Yirga et al., 2018).

### 4.4 ME intake, methane, and heart rate

As in this experiment, in the study of Tsukahara et al. (2016) ruminal methane emission was similar among WT. This suggests that activity and/or level of methanogens were not affected by WT. High dietary concentrations of sulfate can lower methane production in the rumen by acting as a hydrogen sink (Patra and Yu, 2014; Van Zijderveld et al., 2010), but 100-BR and 150-BR in the present experiment did not impact ruminal methanogenesis despite the relatively high sulfate levels. However, sulfate intake (maximum 6.02 g/day equivalent to 0.56% of DM intake for KAT) was much less than that in the study of Van Zijderveld et al. (2010), in which 2.6% of DM intake decreased methane production by 15% (25.5 L/day vs. 21.6 L/day).

The lack of effect of WT on heat energy is somewhat in agreement with inconsistent effects in previous experiments with this brackish water source. In the study of Tsukahara et al. (2016), heat energy in kJ/kg BW^0.75^ was greatest among water treatments for 100% fresh water, suggesting that there was little to no energy cost of excreting the greater quantity of minerals consumed with brackish water inclusion. Also, with similar recovered energy among water treatments, it was concluded that this source of brackish water consumed without addition of other minerals should not adversely affect performance of growing meat goats (Tsukahara et al., 2016). In the study of Yirga et al. (2018), ruminal methane emission was not affected by an increasing level of this brackish water source as well as additional NaCl included with the brackish water in growing or mature Boer goats, but there was a quadratic effect in mature Katahdin sheep as TDS increased with highest methane production at 5600 mg/L. Intake of ME and recovered energy by the sheep decreased with increasing TDS level because of decreasing DM intake but heat energy was not impacted by TDS level, which was postulated as relating to a factor such as decreasing gut motility (Yirga et al., 2018).

It is not clear why increased heart rate only for the 150-BR/SL WT was noted compared with other WT, including 150-BR and 150-SL, despite this WT having levels of all constituents lower than in 150-BR and 150-SL. In a study with goats consuming water with 5000 and 11,000 mg/L of TDS due to NaCl addition, a higher pulse rate was observed for water with a TDS level of 11,000 mg/L than for reservoir water with 33 mg/L TDS and 5000 mg/L TDS (Mdletshe et al., 2017). Also, in lactating Holstein cows, drinking water containing TDS of 3440 mg/kg with 1050 mg/kg of sulfate, 810 mg/kg of sodium, and 250 mg/kg of calcium had elevated heart rate compared with a low TDS water control treatment (Shapasand et al., 2010).

### 4.5 Ruminal fluid characteristics

All ruminal fluid characteristics evaluated were affected by AT. However, it is not possible to identify clear reasons for the differences. Nonetheless, some general considerations can be put forward. The overall higher concentration of total VFA for goats than for sheep could relate to a lower mass of the reticulorumen relative to BW of goats than sheep (NRC, 2007), which presumably is associated with goats being an “intermediate browser” and sheep a “grazer” (NRC, 2007; Van Soest, 1994), although, this postulation entails an assumption that tissue mass relates directly to digesta volume. The increased VFA concentration in goats may also relate to a greater degradation rate of feeds in goats vs. sheep. García et al. (1995) reported a greater DM degradation rate in goats than in sheep when the pasture selected was high in cell wall content, similar to the feeding conditions of the present study. The difference in total VFA is despite DM intake by % BW being or tending to be less for goats vs. sheep, along with generally similar OM digestibility. The VFA difference is in accordance with lower ruminal pH for goats than for sheep.

Factors responsible for significant but relatively small differences in molar percentages of individual VFA are unclear, as is also the case for the AT × WT interaction in the level of butyrate. The only effect of WT occurred with KAT, and magnitudes of difference among treatments were relatively small. Water salinity in a natural source also did not influence ruminal fermentation conditions with TDS up to 8000 mg/L in buffalo calves (Preeti et al., 2018) and 9220 mg/L by addition of different salts, resulting in a sodium concentration of 2770 mg/L and sulfate of 2090 mg/L in Holstein cows (Valtorta et al., 2008). Total numbers of amylolytic and cellulolytic bacteria and protozoal populations were not affected by water with a TDS level up to 9220 mg/L (Valtorta et al., 2008).

The lower ruminal ammonia nitrogen concentration for ANG vs. BOE and SPA could involve low DM intake (i.e., % BW) for ANG. With similar DM intake relative to BW and nitrogen digestion for BOE and SPA, the greater ammonia nitrogen level for BOE could involve a difference in ruminal ammonia nitrogen recycling and absorption and/or microbial protein synthesis. The highest urea nitrogen concentration among AT for BOE is in accordance with the relatively high level of ammonia nitrogen in ruminal fluid, perhaps accompanied by relatively high urea diffusion because of a large gradient between blood and rumen wall-adherent urease-producing bacteria (Cheng and Costerton, 1980).

### 4.6 Blood constituent levels

There were effects of AT on many blood measures. Higher PCV values for sheep than for goats was also observed by Tsukahara et al. (2021), which was thought to be because of smaller erythrocytes in goats that allow for tighter packing with centrifugation (Smith and Sherman, 2009). Factors responsible for the lower PCV for BOE than for ANG and SPA are unclear. However, this is in agreement with the relatively low hemoglobin concentration for BOE than for ANG and SPA, which contributed to the lowest blood oxygen concentration among AT for BOE. Relatedly, the higher concentration of hemoglobin for sheep vs. goats in accordance with the PCV difference resulted in a greater blood oxygen concentration for sheep. Physiological implications of this difference are unclear, since only venous jugular blood was sampled and analyzed.

The lack of interaction between AT and WT in osmolality reflects a similar capacity of these AT for regulation via water intake, urine output, and excretion of minerals from consumption of brackish water with and without added NaCl. Animals within physiological limits maintain osmolality and acceptable levels of electrolytes by regulating filtration in the kidneys. All breeds of sheep and goats decreased aldosterone concentration in blood when water with elevated TDS levels was consumed to keep osmolarity and electrolytes in balance. Similar osmolarity among AT perhaps reflects tight regulation of blood sodium and chloride concentrations. However, there were differences among AT in concentrations of cations of calcium, potassium, and magnesium. Although, it is unclear why calcium and potassium concentrations were higher for sheep relative to goats, as is true for the relatively low magnesium concentration for ANG. Similar to this study, breed differences among Holstein Friesian, Hereford, Simmental, and Limousin cattle in plasma mineral (i.e., calcium, chloride, sodium and potassium) concentrations were observed by Mordak et al. (2007).

The lowest concentration of glucose among AT for ANG probably relates to low DM intake (% BW), although factors responsible for relatively high levels for DOR and STC are unclear. Likewise, the cause of the very high level of lactate for DOR is unknown. Why the concentration of albumin was lowest among AT for BOE is unclear as well. Though the concentration of triglycerides was similar among AT, the highest level of cholesterol for ANG may again be linked to relatively low DM intake (% BW) that suggests relatively high adipose tissue mobilization.

Differences among AT in the concentration of each enzyme measured reflect conditions such as body composition and physiological processes in multiple organs and tissues. Although the concentration of alkaline phosphatase, mostly released from liver and bone (Lowe et al., 2022), was markedly greater for SPA than for other AT, it was within a range of 93 to 387 U/L vs. 298 U/L proposed for goats (Merck Veterinary Manual, 2022). Similar to this study, differences among breeds in plasma aspartate transferase (Holstein Friesian vs. Hereford, Simmental, and Limousine cattle, Mordak et al., 2007; Arsi-bale vs. Long-eared Somali vs. Central Highland goats, Tibbo et al., 2008), creatinine (Karakachan vs. Copper-Red Shumen sheep, Angelov et al., 2013; Hereford vs. Limousin cattle, Mordak et al., 2007), creatine kinase activity (Dorper vs. Blackhead Persian, Dorper, and South African Mutton Merino, Chulayo and Muchenje, 2013), and gamma glutamyl transferase (Texel vs. Flemish Milk and Finnish Landrace sheep, Van der Berg et al., 1983) have been reported. The activity of aspartate transferase that arises primarily from the liver and that of gamma glutamyl transferase mainly expressed in kidneys and the liver are used to evaluate liver and kidney function. Creatinine, creatine phosphate, and creatine kinase are involved in the rapid supply of ATP, and creatine is the metabolite of creatinine, which all relate to body mass, muscle protein breakdown, and kidney function (Lérias et al., 2015). Increased creatinine kinase activity of ANG with some WT (FR vs. 100-BR and 100-SL) and BOE (FR and 100-BR vs. 150-BR), especially for FR, is unclear in regard to differences in mineral levels in the WT. Nonetheless, an inverse correlation between urinary sodium excretion and creatine kinase has been reported, and high creatine kinase activity in the kidneys may increase ATP availability for active sodium resorption (Brewster et al., 2018; Pisoni et al., 2018).

Total antioxidant balance in plasma is influenced by formation of reactive oxygen species and combined effects of many different oxidants and antioxidant compounds including vitamins E and C, carotene, glutathione, taurine, and various enzymes (Quijano et al., 2016; Sardesai, 1995). Heat energy relative to BW^0.75^ was lower in ANG than other AT in this study. Therefore, the relatively high total antioxidant capacity for ANG may reflect lower heat energy associated with limited feed intake, as energy metabolism is associated with generation of reactive oxygen species (Quijano et al., 2016). Animal breed or cluster (i.e., group of breeds) differences in antioxidant status were also noted in other studies (Kirschvink et al., 2006; Manuelian et al., 2020). Considerable variability in the aldosterone concentration resulted in similar values among AT, although numerically the value for BOE was much higher (approximately 2 times) than for other AT. Breed differences in aldosterone concentration have been observed, examples being St. Croix vs. Dorper and Katahdin sheep (Hussein et al., 2022) and Osmanabadi vs. Malabari and Salem black goats (Aleena et al., 2020).

As noted before, WT had very few effects, as is the case for concentrations of blood constituents except aldosterone. A relatively high aldosterone concentration for FR presumably relates to elevated concentrations of TDS in brackish water treatments because of high levels of sodium. The higher concentration of aldosterone for FR would have allowed for increased sodium absorption from the kidneys to maintain an acceptable level of sodium and osmolality in the blood. However, that there were no differences among WT with inclusion of brackish water or added NaCl may indicate that differences in sodium were not high enough (ranging 1387 to 2698 mg/kg; 2 times higher) to affect aldosterone concentration relative to the difference between FR and other treatments (16.9 to 32.5 times greater). Aldosterone-regulated sodium transport in kidney tubules seems highly efficient for homeostatic mechanisms of sodium and osmolality within certain ranges (Meneton et al., 2004; Rossi et al., 2020).

## 5 Conclusion

It was hypothesized that breeds of sheep and goats would be affected differently by WT varying in levels and types of minerals from the brackish water source and additions of NaCl. Although there were numerous differences among AT, there were very few significant effects of AT or interactions between WT and AT, and ones noted did not seem of great relevance. Thus, the breeds of yearling sheep (Dorper, Katahdin, and St. Croix ewe lambs) and goats (Angora, Boer, and Spanish doelings) used in this short-term study seemed resilient to the WT regardless of mineral source and levels with TDS less than 8300 mg/kg. However, longer term studies with evaluation of performance and health would be beneficial regarding potential for reliance on such sources of drinking water by small ruminants in common production settings.

## Author contributions

**Amlan Kumar Patra:** Validation, Formal analysis, Writing—original draft preparation, Writing—review and editing. **Luana Paula dos Santos Ribeiro:** Methodology, Validation, Formal analysis, Investigation, Data curation, Writing—original draft preparation, Writing—review and editing. **Hirut** **Yirga** and **Adekayode O. Sonibare:** Writing—original draft preparation, Writing—review and editing. **Ahmed R. Askar** and **Ali H. Hussein:** Writing—original draft preparation. **Ryszard Puchala:** Conceptualization, Methodology, Validation, Formal analysis, Investigation, Resources, Data curation, Writing—original draft preparation, Writing—review and editing, Supervision, Funding acquisition. **Arthur Louis Goetsch:** Conceptualization, Methodology, Validation, Formal analysis, Investigation, Resources, Data curation, Writing—original draft preparation, Writing—review and editing, Supervision, Project administration, Funding acquisition.

## Declaration of competing interest

We declare that we have no financial and personal relationships with other people or organizations that can inappropriately influence our work, and there is no professional or other personal interest of any nature or kind in any product, service and/or company that could be construed as influencing the content of this paper.

## Data availability

All data generated from this study are presented in the tables.
