# Supplementary material for: Effects of the concentration and nature of total dissolved solids in drinking water on feed intake, nutrient digestion, energy balance, methane emission, ruminal fermentation, and blood constituents in different breeds of young goats and hair sheep
Source: Anim Nutr. 2023 Oct 30;16:84–95. doi: 10.1016/j.aninu.2023.10.002 (PMC10851211; doi:10.1016/j.aninu.2023.10.002)
Supplement: Multimedia component 1 [file mmc1.docx]

| Table S1. Effects of water treatment and animal type × water treatment on body weight and dry matter and water intake during the 3-wk periods of yearling goat doelings and hair sheep ewe lambs. | | | | | | | | |
| --- | --- | --- | --- | --- | --- | --- | --- | --- |
| Item | Animal type^1^ | Water treatment^2^ | | | | | | SEM^3^ |
|  |  | FR | 100-BR | 100-SL | 150-BR | 150-SL | 150-BR/SL |  |
| Body weight, kg | Mean | 26.3 | 26.3 | 26.2 | 26.2 | 26.4 | 26.2 | 0.58 |
|  | ANG | 16.1 | 16.9 | 17.4 | 18.0 | 17.3 | 16.3 | 1.39 |
|  | BOE | 20.7 | 20.3 | 20.0 | 20.4 | 20.4 | 20.8 |  |
|  | SPA | 17.8 | 17.4 | 17.2 | 17.5 | 17.5 | 17.7 |  |
|  | DOR | 38.5 | 38.2 | 37.8 | 37.4 | 38.3 | 38.6 |  |
|  | KAT | 34.2 | 34.7 | 34.4 | 34.3 | 34.6 | 33.9 |  |
|  | STC | 30.6 | 30.4 | 30.1 | 29.5 | 30.1 | 30.2 |  |
| Dry matter |  |  |  |  |  |  |  |  |
| Intake, g/day | Mean | 703 | 709 | 701 | 732 | 736 | 739 | 32.9 |
|  | ANG | 303 | 330 | 297 | 410 | 383 | 385 | 78.2 |
|  | BOE | 551 | 500 | 484 | 561 | 498 | 527 |  |
|  | SPA | 471 | 410 | 486 | 446 | 483 | 432 |  |
|  | DOR | 1000 | 991 | 1057 | 1025 | 1089 | 1107 |  |
|  | KAT | 1020 | 1037 | 938 | 1078 | 992 | 1057 |  |
|  | STC | 873 | 988 | 943 | 877 | 973 | 924 |  |
| Intake, % body weight | Mean | 2.58 | 2.57 | 2.60 | 2.73 | 2.71 | 2.73 | 0.103 |
|  | ANG | 1.86 | 1.90 | 1.68 | 2.26 | 2.14 | 2.38 | 0.248 |
|  | BOE | 2.67 | 2.45 | 2.42 | 2.72 | 2.45 | 2.55 |  |
|  | SPA | 2.59 | 2.37 | 2.80 | 2.55 | 2.75 | 2.43 |  |
|  | DOR | 2.58 | 2.57 | 2.81 | 2.75 | 2.81 | 2.81 |  |
|  | KAT | 2.97 | 2.96 | 2.80 | 3.15 | 2.88 | 3.16 |  |
|  | STC | 2.81 | 3.21 | 3.11 | 2.96 | 3.23 | 3.05 |  |
| Water |  |  |  |  |  |  |  |  |
| Intake, g/day | ANG | 876 | 841 | 736 | 916 | 893 | 967 | 171.0 |
|  | BOE | 750 | 926 | 806 | 834 | 978 | 672 |  |
|  | SPA | 722 | 620 | 630 | 492 | 801 | 827 |  |
|  | DOR | 1702 | 1395 | 1797 | 1601 | 2209 | 1946 |  |
|  | KAT | 1995 | 1811 | 1935 | 1974 | 1995 | 1955 |  |
|  | STC | 1337 | 1520 | 1560 | 1310 | 1689 | 1475 |  |
| Intake, % body weight | ANG | 5.65 | 4.83 | 4.30 | 5.09 | 5.31 | 6.04 | 0.605 |
|  | BOE | 3.65 | 4.48 | 4.05 | 4.07 | 4.81 | 3.29 |  |
|  | SPA | 4.09 | 3.61 | 3.65 | 2.71 | 4.55 | 4.65 |  |
|  | DOR | 4.41 | 3.67 | 4.80 | 4.28 | 5.72 | 5.85 |  |
|  | KAT | 5.80 | 5.32 | 5.66 | 5.82 | 5.72 | 5.85 |  |
|  | STC | 4.39 | 5.10 | 5.23 | 4.52 | 5.68 | 4.88 |  |
| Intake, g/g dry matter | Mean | 2.02 | 1.81 | 1.90 | 1.66 | 2.03 | 1.88 | 0.119 |
|  | ANG | 3.66 | 2.64 | 2.73 | 2.33 | 2.75 | 2.72 | 0.289 |
|  | BOE | 1.37 | 1.78 | 1.71 | 1.49 | 1.98 | 1.30 |  |
|  | SPA | 1.73 | 1.50 | 1.29 | 1.10 | 1.69 | 1.91 |  |
|  | DOR | 1.82 | 1.48 | 1.71 | 1.62 | 1.95 | 1.84 |  |
|  | KAT | 1.98 | 1.83 | 2.23 | 1.85 | 2.03 | 1.89 |  |
|  | STC | 1.57 | 1.61 | 1.74 | 1.54 | 1.79 | 1.64 |  |
| ^1^ANG = Angora goats; BOE = Boer goats; SPA = Spanish goats; DOR = Dorper sheep; KAT = Katahdin sheep; STC = St. Croix sheep.  ^2^FR = fresh water; 100-BR = brackish water (BR); 100-SL = NaCl added to FR for a similar total dissolved solids (TDS) level as 100-BR; 150-BR = 100-BR plus addition of approximately 50% of 100-BR TDS; 150-SL = NaCl added to FR for a similar TDS level as 150-BR; 150-BR/SL = a similar TDS level as 150-BR and 150-SL by addition to 100-BR of a 1:1 mixture of BR minerals and NaCl.  ^3^SEM = pooled standard error of the mean for the water treatment effect (mean values) or interaction between water treatment and animal type. | | | | | | | | |

| Table S2. Effects of water treatment and animal type × water treatment on intake during the fecal collection period and digestion measures of yearling goat doelings and hair sheep ewe lambs. | | | | | | | | |
| --- | --- | --- | --- | --- | --- | --- | --- | --- |
| Item | Animal type^1^ | Water treatment^2^ | | | | | | SEM^3^ |
|  |  | FR | 100-BR | 100-SL | 150-BR | 150-SL | 150-BR/SL |  |
| Dry matter |  |  |  |  |  |  |  |  |
| Intake, g/day | Mean | 743 | 747 | 754 | 761 | 773 | 756 | 31.2 |
|  | ANG | 319 | 317 | 318 | 424 | 417 | 423 | 76.6 |
|  | BOE | 582 | 505 | 536 | 629 | 538 | 551 |  |
|  | SPA | 524 | 474 | 512 | 483 | 540 | 451 |  |
|  | DOR | 1022 | 1034 | 1119 | 1052 | 1095 | 1075 |  |
|  | KAT | 1115 | 1139 | 1050 | 1092 | 1090 | 1077 |  |
|  | STC | 895 | 1011 | 993 | 883 | 957 | 959 |  |
| Digestion, % | Mean | 62.4 | 63.2 | 63.6 | 61.2 | 64.8 | 61.6 | 1.17 |
|  | ANG | 54.7 | 57.9 | 60.6 | 62.2 | 63.8 | 56.1 | 2.86 |
|  | BOE | 65.2 | 64.1 | 62.1 | 54.5 | 63.4 | 65.6 |  |
|  | SPA | 61.8 | 62.5 | 61.2 | 64.0 | 66.5 | 60.9 |  |
|  | DOR | 63.8 | 64.9 | 64.3 | 65.4 | 65.8 | 60.4 |  |
|  | KAT | 65.1 | 67.3 | 65.3 | 66.9 | 67.2 | 65.2 |  |
|  | STC | 64.0 | 62.4 | 68.1 | 54.4 | 62.2 | 61.7 |  |
| Digested, g/day | Mean | 473 | 481 | 489 | 474 | 506 | 473 | 23.7 |
|  | ANG | 177 | 192 | 199 | 265 | 268 | 248 | 58.1 |
|  | BOE | 383 | 322 | 333 | 346 | 342 | 359 |  |
|  | SPA | 327 | 296 | 315 | 311 | 360 | 277 |  |
|  | DOR | 650 | 679 | 720 | 688 | 729 | 662 |  |
|  | KAT | 726 | 772 | 690 | 730 | 731 | 704 |  |
|  | STC | 577 | 627 | 677 | 505 | 607 | 590 |  |
| Organic matter |  |  |  |  |  |  |  |  |
| Intake, g/day | Mean | 676 | 679 | 685 | 692 | 703 | 687 | 28.4 |
|  | ANG | 289 | 288 | 289 | 386 | 378 | 384 | 69.5 |
|  | BOE | 529 | 459 | 482 | 572 | 489 | 501 |  |
|  | SPA | 477 | 431 | 466 | 439 | 491 | 410 |  |
|  | DOR | 930 | 941 | 1018 | 957 | 996 | 978 |  |
|  | KAT | 1014 | 1036 | 955 | 993 | 991 | 979 |  |
|  | STC | 814 | 919 | 904 | 803 | 870 | 872 |  |
| Digestion, % | Mean | 65.3 | 66.0 | 66.4 | 64.3 | 67.5 | 64.6 | 1.08 |
|  | ANG | 57.5 | 61.0 | 63.3 | 64.9 | 66.4 | 59.1 | 2.64 |
|  | BOE | 67.9 | 67.0 | 65.0 | 58.2 | 66.1 | 68.2 |  |
|  | SPA | 64.3 | 65.0 | 63.5 | 66.5 | 68.7 | 63.4 |  |
|  | DOR | 66.8 | 67.8 | 67.5 | 68.4 | 68.7 | 63.7 |  |
|  | KAT | 68.3 | 69.9 | 68.5 | 69.9 | 70.0 | 68.3 |  |
|  | STC | 66.9 | 65.5 | 70.6 | 58.2 | 65.3 | 65.0 |  |
| Digested, g/day | Mean | 450 | 457 | 464 | 453 | 480 | 451 | 22.1 |
|  | ANG | 168 | 183 | 188 | 251 | 253 | 236 | 54.0 |
|  | BOE | 362 | 306 | 316 | 336 | 324 | 340 |  |
|  | SPA | 309 | 280 | 297 | 294 | 338 | 262 |  |
|  | DOR | 619 | 644 | 687 | 654 | 692 | 634 |  |
|  | KAT | 693 | 729 | 657 | 695 | 692 | 670 |  |
|  | STC | 548 | 599 | 639 | 488 | 579 | 566 |  |
| Neutral detergent fiber |  |  |  |  |  |  |  |  |
| Intake, g/day | Mean | 493 | 496 | 501 | 505 | 513 | 502 | 20.8 |
|  | ANG | 210 | 209 | 210 | 281 | 275 | 280 | 50.9 |
|  | BOE | 386 | 334 | 351 | 417 | 357 | 365 |  |
|  | SPA | 348 | 314 | 339 | 320 | 358 | 298 |  |
|  | DOR | 680 | 688 | 744 | 700 | 728 | 715 |  |
|  | KAT | 742 | 758 | 698 | 726 | 725 | 716 |  |
|  | STC | 595 | 672 | 661 | 589 | 636 | 638 |  |
| Digestion, % | Mean | 67.0 | 67.3 | 67.4 | 65.5 | 68.7 | 66.4 | 1.08 |
|  | ANG | 58.8 | 60.6 | 63.4 | 65.3 | 67.3 | 60.6 | 2.64 |
|  | BOE | 69.2 | 69.7 | 66.9 | 59.6 | 68.5 | 70.7 |  |
|  | SPA | 66.5 | 66.6 | 64.7 | 67.2 | 69.6 | 65.5 |  |
|  | DOR | 68.5 | 68.9 | 67.9 | 70.2 | 69.6 | 64.8 |  |
|  | KAT | 70.1 | 70.3 | 69.7 | 71.1 | 71.0 | 69.8 |  |
|  | STC | 68.9 | 67.5 | 71.9 | 59.8 | 66.2 | 67.1 |  |
| Digested, g/day | Mean | 337 | 340 | 344 | 337 | 356 | 338 | 16.4 |
|  | ANG | 125 | 131 | 137 | 184 | 187 | 175 | 40.1 |
|  | BOE | 268 | 232 | 235 | 250 | 245 | 257 |  |
|  | SPA | 233 | 209 | 221 | 216 | 245 | 197 |  |
|  | DOR | 463 | 478 | 506 | 490 | 512 | 473 |  |
|  | KAT | 522 | 535 | 490 | 517 | 514 | 502 |  |
|  | STC | 412 | 454 | 477 | 366 | 429 | 426 |  |
| Nitrogen |  |  |  |  |  |  |  |  |
| Intake, g/day | Mean | 12.0 | 12.1 | 12.0 | 12.3 | 12.4 | 12.2 | 0.60 |
|  | ANG | 5.3 | 5.2 | 4.2 | 7.0 | 6.8 | 6.9 | 1.22 |
|  | BOE | 9.5 | 8.2 | 8.6 | 10.2 | 8.4 | 9.0 |  |
|  | SPA | 8.5 | 7.7 | 8.4 | 7.9 | 8.8 | 7.4 |  |
|  | DOR | 16.5 | 16.7 | 18.0 | 16.9 | 17.6 | 17.3 |  |
|  | KAT | 18.0 | 18.3 | 16.9 | 17.6 | 17.5 | 17.3 |  |
|  | STC | 14.5 | 16.3 | 16.0 | 14.3 | 15.4 | 15.5 |  |
| Digestion, % | Mean | 62.8 | 62.6 | 64.4 | 61.0 | 65.3 | 61.8 | 1.30 |
|  | ANG | 56.1 | 59.4 | 66.7 | 65.1 | 64.9 | 57.6 | 3.16 |
|  | BOE | 65.4 | 63.5 | 63.0 | 54.9 | 65.4 | 67.0 |  |
|  | SPA | 62.7 | 62.5 | 63.7 | 64.7 | 67.1 | 61.8 |  |
|  | DOR | 63.0 | 64.7 | 61.9 | 64.9 | 65.6 | 59.6 |  |
|  | KAT | 65.5 | 65.7 | 64.5 | 67.0 | 66.6 | 65.2 |  |
|  | STC | 64.3 | 59.6 | 66.5 | 49.6 | 62.3 | 59.5 |  |
| Digested, g/day | Mean | 7.7 | 7.6 | 7.8 | 7.5 | 8.2 | 7.6 | 0.39 |
|  | ANG | 3.1 | 3.3 | 2.9 | 4.5 | 4.7 | 4.2 | 1.02 |
|  | BOE | 6.3 | 5.2 | 5.5 | 5.5 | 5.5 | 6.0 |  |
|  | SPA | 5.4 | 4.8 | 5.7 | 5.2 | 5.9 | 4.6 |  |
|  | DOR | 10.3 | 10.8 | 11.2 | 10.9 | 11.7 | 10.4 |  |
|  | KAT | 11.7 | 12.1 | 10.9 | 11.7 | 11.7 | 11.3 |  |
|  | STC | 9.3 | 9.6 | 10.7 | 7.2 | 9.7 | 9.1 |  |
| ^1^ANG = Angora goats; BOE = Boer goats; SPA = Spanish goats; DOR = Dorper sheep; KAT = Katahdin sheep; STC = St. Croix sheep.  ^2^FR = fresh water; 100-BR = brackish water (BR); 100-SL = NaCl added to FR for a similar total dissolved solids (TDS) level as 100-BR; 150-BR = 100-BR plus addition of approximately 50% of 100-BR TDS; 150-SL = NaCl added to FR for a similar TDS level as 150-BR; 150-BR/SL = a similar TDS level as 150-BR and 150-SL by addition to 100-BR of a 1:1 mixture of BR minerals and NaCl.  ^3^SEM = pooled standard error of mean for the water treatment effect (mean values) or interaction between water treatment and animal type. | | | | | | | | |

| Table S3. Effects of water treatment and animal type × water treatment on measures during the calorimetry period of yearling goat doelings and hair sheep ewe lambs. | | | | | | | | |
| --- | --- | --- | --- | --- | --- | --- | --- | --- |
| Item | Animal type^1^ | Water treatment^2^ | | | | | | SEM^3^ |
|  |  | FR | 100-BR | 100-SL | 150-BR | 150-SL | 150-BR/SL |  |
| Dry matter intake, g/day | Mean | 633 | 668 | 675 | 705 | 647 | 704 | 37.9 |
|  | ANG | 232 | 244 | 217 | 390 | 351 | 359 | 92.3 |
|  | BOE | 524 | 488 | 429 | 611 | 436 | 482 |  |
|  | SPA | 366 | 340 | 430 | 389 | 457 | 385 |  |
|  | DOR | 911 | 1050 | 1034 | 956 | 1028 | 1018 |  |
|  | KAT | 1012 | 1060 | 1080 | 1063 | 792 | 1043 |  |
|  | STC | 754 | 823 | 861 | 819 | 815 | 934 |  |
| Gross energy intake, MJ/day | Mean | 11.12 | 11.73 | 11.86 | 12.38 | 11.36 | 12.36 | 0.670 |
|  | ANG | 4.06 | 4.28 | 3.80 | 6.84 | 16.17 | 6.30 | 1.624 |
|  | BOE | 9.20 | 8.57 | 7.54 | 10.73 | 7.66 | 8.47 |  |
|  | SPA | 6.42 | 5.97 | 7.54 | 6.84 | 8.03 | 6.76 |  |
|  | DOR | 16.01 | 18.46 | 18.18 | 16.81 | 18.07 | 17.90 |  |
|  | KAT | 17.81 | 18.64 | 18.99 | 18.70 | 13.92 | 18.34 |  |
|  | STC | 13.25 | 14.46 | 15.13 | 14.39 | 14.33 | 16.41 |  |
| Digestible energy intake, MJ/day | Mean | 7.43 | 7.92 | 8.06 | 8.09 | 7.76 | 8.13 | 0.491 |
|  | ANG | 2.34 | 2.70 | 2.49 | 4.47 | 4.14 | 3.81 | 1.195 |
|  | BOE | 6.33 | 5.69 | 4.93 | 6.29 | 5.15 | 5.79 |  |
|  | SPA | 4.16 | 3.88 | 4.81 | 4.61 | 5.50 | 4.33 |  |
|  | DOR | 10.68 | 12.70 | 12.30 | 11.46 | 12.46 | 11.52 |  |
|  | KAT | 12.12 | 13.10 | 13.13 | 13.10 | 9.73 | 12.62 |  |
|  | STC | 8.92 | 9.45 | 10.70 | 8.62 | 9.56 | 10.71 |  |
| Heart rate, beats/min | Mean | 78.2^a^ | 81.2^a^ | 80.6^a^ | 78.5^a^ | 79.3^a^ | 86.1^b^ | 2.07 |
|  | ANG | 64.5 | 68.2 | 82.6 | 71.9 | 76.0 | 89.7 | 5.04 |
|  | BOE | 81.0 | 92.0 | 83.3 | 81.5 | 81.9 | 88.5 |  |
|  | SPA | 80.5 | 83.5 | 86.5 | 77.8 | 85.3 | 96.2 |  |
|  | DOR | 81.7 | 78.7 | 80.0 | 77.5 | 78.0 | 81.5 |  |
|  | KAT | 80.0 | 81.7 | 79.2 | 80.7 | 76.7 | 80.2 |  |
|  | STC | 81.3 | 83.2 | 74.5 | 81.5 | 77.8 | 80.3 |  |
| Respiratory quotient | Mean | 0.948 | 0.953 | 0.951 | 0.960 | 0.961 | 0.965 | 0.0078 |
|  | ANG | 0.883 | 0.869 | 0.878 | 0.878 | 0.891 | 0.901 | 0.0189 |
|  | BOE | 0.946 | 0.924 | 0.930 | 0.979 | 0.981 | 0.951 |  |
|  | SPA | 0.901 | 0.911 | 0.932 | 0.945 | 0.948 | 0.942 |  |
|  | DOR | 0.979 | 1.003 | 0.983 | 0.958 | 0.992 | 1.000 |  |
|  | KAT | 0.998 | 0.999 | 1.012 | 0.999 | 0.991 | 0.983 |  |
|  | STC | 0.979 | 1.011 | 0.979 | 0.999 | 0.962 | 1.012 |  |
| Heat energy, MJ/day | Mean | 4.76 | 4.88 | 4.77 | 4.96 | 4.96 | 4.97 | 0.138 |
|  | ANG | 2.63 | 2.63 | 2.40 | 3.14 | 2.97 | 3.05 | 0.337 |
|  | BOE | 3.79 | 3.86 | 3.66 | 3.93 | 3.63 | 3.75 |  |
|  | SPA | 3.16 | 3.18 | 3.41 | 3.29 | 3.33 | 3.33 |  |
|  | DOR | 6.96 | 7.05 | 7.20 | 6.77 | 7.06 | 7.33 |  |
|  | KAT | 6.36 | 6.40 | 6.28 | 6.75 | 6.75 | 6.66 |  |
|  | STC | 5.65 | 6.17 | 5.68 | 5.91 | 5.98 | 5.68 |  |
| Heat energy, kJ/kg BW^0.75^ | Mean | 407 | 417 | 406 | 420 | 415 | 421 | 7.8 |
|  | ANG | 343 | 332 | 314 | 365 | 364 | 375 | 19.1 |
|  | BOE | 405 | 402 | 388 | 409 | 383 | 393 |  |
|  | SPA | 362 | 387 | 399 | 390 | 396 | 393 |  |
|  | DOR | 445 | 460 | 461 | 435 | 446 | 468 |  |
|  | KAT | 446 | 460 | 444 | 473 | 465 | 466 |  |
|  | STC | 438 | 462 | 430 | 447 | 434 | 431 |  |
| Heat energy, % DE intake | Mean | 78 | 80 | 76 | 80 | 71 | 72 | 5.8 |
|  | ANG | 114 | 132 | 121 | 87 | 78 | 96 | 13.8 |
|  | BOE | 75 | 79 | 89 | 95 | 71 | 67 |  |
|  | SPA | 90 | 87 | 74 | 98 | 61 | 87 |  |
|  | DOR | 70 | 64 | 60 | 63 | 59 | 68 |  |
|  | KAT | 54 | 53 | 57 | 55 | 78 | 57 |  |
|  | STC | 66 | 67 | 55 | 79 | 81 | 57 |  |
| Methane energy, MJ/day | Mean | 0.78 | 0.84 | 0.81 | 0.84 | 0.85 | 0.85 | 0.037 |
|  | ANG | 0.25 | 0.30 | 0.22 | 0.40 | 0.35 | 0.42 | 0.092 |
|  | BOE | 0.59 | 0.62 | 0.62 | 0.67 | 0.61 | 0.62 |  |
|  | SPA | 0.44 | 0.42 | 0.48 | 0.47 | 0.52 | 0.51 |  |
|  | DOR | 1.19 | 1.22 | 1.33 | 1.16 | 1.33 | 1.31 |  |
|  | KAT | 1.24 | 1.20 | 1.15 | 1.24 | 1.22 | 1.19 |  |
|  | STC | 0.98 | 1.29 | 1.06 | 1.11 | 1.10 | 1.03 |  |
| Methane energy, % GE intake | Mean | 7.17 | 7.62 | 7.21 | 7.18 | 7.68 | 7.20 | 0.369 |
|  | ANG | 6.45 | 7.63 | 6.27 | 5.92 | 6.18 | 7.21 | 0.898 |
|  | BOE | 7.15 | 8.10 | 8.90 | 6.45 | 8.23 | 7.53 |  |
|  | SPA | 7.22 | 7.02 | 6.56 | 8.61 | 6.31 | 7.72 |  |
|  | DOR | 7.75 | 7.19 | 7.41 | 7.10 | 7.59 | 7.58 |  |
|  | KAT | 6.97 | 6.77 | 6.99 | 7.01 | 9.48 | 6.64 |  |
|  | STC | 7.47 | 9.00 | 7.13 | 8.00 | 8.30 | 6.49 |  |
| Methane energy, % DE intake | Mean | 11.11 | 11.60 | 11.01 | 12.45 | 11.44 | 11.32 | 0.830 |
|  | ANG | 11.59 | 12.27 | 10.41 | 9.28 | 9.42 | 12.38 | 2.018 |
|  | BOE | 10.67 | 12.10 | 13.79 | 16.87 | 12.16 | 11.12 |  |
|  | SPA | 11.31 | 10.88 | 10.37 | 13.31 | 9.22 | 12.50 |  |
|  | DOR | 11.66 | 10.78 | 11.03 | 10.38 | 11.00 | 11.91 |  |
|  | KAT | 10.20 | 9.74 | 10.33 | 10.04 | 13.92 | 9.84 |  |
|  | STC | 11.21 | 13.82 | 12.13 | 14.82 | 12.90 | 10.16 |  |
| Urine energy, MJ/day | Mean | 0.15 | 0.18 | 0.17 | 0.18 | 0.17 | 0.18 | 0.012 |
|  | ANG | 0.10 | 0.08 | 0.08 | 0.11 | 0.10 | 0.09 | 0.029 |
|  | BOE | 0.12 | 0.16 | 0.13 | 0.16 | 0.13 | 0.16 |  |
|  | SPA | 0.10 | 0.10 | 0.09 | 0.10 | 0.11 | 0.12 |  |
|  | DOR | 0.21 | 0.32 | 0.26 | 0.24 | 0.25 | 0.26 |  |
|  | KAT | 0.23 | 0.22 | 0.28 | 0.25 | 0.21 | 0.25 |  |
|  | STC | 0.16 | 0.18 | 0.15 | 0.19 | 0.19 | 0.19 |  |
| Metabolizable energy intake, MJ/day | Mean | 6.49 | 6.90 | 7.09 | 7.07 | 6.73 | 7.11 | 0.469 |
|  | ANG | 2.00 | 2.32 | 2.20 | 3.96 | 3.69 | 3.31 | 1.112 |
|  | BOE | 5.62 | 4.90 | 4.17 | 5.46 | 4.42 | 5.01 |  |
|  | SPA | 3.62 | 3.37 | 4.24 | 4.05 | 4.88 | 3.70 |  |
|  | DOR | 9.27 | 11.16 | 10.11 | 10.06 | 10.87 | 9.95 |  |
|  | KAT | 10.65 | 11.67 | 11.70 | 11.60 | 8.30 | 11.18 |  |
|  | STC | 7.78 | 7.98 | 9.49 | 7.32 | 8.27 | 9.49 |  |
| Recovered energy, MJ/day | Mean | 1.73 | 2.02 | 2.31 | 2.11 | 1.79 | 2.14 | 0.409 |
|  | ANG | -0.63 | -0.31 | -0.22 | 0.82 | 0.72 | 0.26 | 0.986 |
|  | BOE | 1.84 | 1.04 | 0.52 | 1.53 | 0.80 | 1.26 |  |
|  | SPA | 0.47 | 0.19 | 0.83 | 0.76 | 1.55 | 0.37 |  |
|  | DOR | 2.31 | 4.11 | 3.51 | 3.29 | 3.81 | 2.63 |  |
|  | KAT | 4.29 | 5.28 | 5.42 | 4.85 | 1.55 | 4.53 |  |
|  | STC | 2.14 | 1.81 | 3.81 | 1.40 | 2.28 | 3.81 |  |
| GE = gross energy; DE = digestible energy.  ^1^ANG = Angora goats; BOE = Boer goats; SPA = Spanish goats; DOR = Dorper sheep; KAT = Katahdin sheep; STC = St. Croix sheep.  ^2^FR = fresh water; 100-BR = brackish water (BR); 100-SL = NaCl added to FR for a similar total dissolved solids (TDS) level as 100-BR; 150-BR = 100-BR plus addition of approximately 50% of 100-BR TDS; 150-SL = NaCl added to FR for a similar TDS level as 150-BR; 150-BR/SL = a similar TDS level as 150-BR and 150-SL by addition to 100-BR of a 1:1 mixture of BR minerals and NaCl.  ^3^SEM = pooled standard error of the mean for the water treatment effect (mean values) or interaction between water treatment and animal type. | | | | | | | | |

^a,b^Means within grouping without a common superscript letter differ (*P <* 0.05).

| Table S4. Effects of water treatment and animal type × water treatment on ruminal fluid characteristics of yearling goat doelings and hair sheep ewe lambs. | | | | | | | | |
| --- | --- | --- | --- | --- | --- | --- | --- | --- |
| Item | Animal type^1^ | Water treatment^2^ | | | | | | SEM^3^ |
|  |  | FR | 100-BR | 100-SL | 150-BR | 150-SL | 150-BR/SL |  |
| pH | Mean | 5.98 | 5.98 | 6.00 | 5.97 | 5.91 | 5.95 | 0.034 |
|  | ANG | 5.77 | 5.91 | 6.00 | 5.82 | 5.73 | 5.81 | 0.083 |
|  | BOE | 5.80 | 5.86 | 5.80 | 5.83 | 5.89 | 5.88 |  |
|  | SPA | 5.87 | 5.84 | 5.84 | 5.88 | 5.75 | 5.74 |  |
|  | DOR | 6.08 | 6.10 | 6.13 | 6.01 | 5.97 | 6.05 |  |
|  | KAT | 6.24 | 6.00 | 6.19 | 6.03 | 6.09 | 6.14 |  |
|  | STC | 6.10 | 6.19 | 6.02 | 6.26 | 6.04 | 6.07 |  |
| Ammonia nitrogen, mg/dL | Mean | 7.1 | 6.8 | 7.5 | 7.5 | 7.0 | 6.8 | 0.40 |
|  | ANG | 4.2 | 6.8 | 6.7 | 7.2 | 6.2 | 4.8 | 0.96 |
|  | BOE | 11.2 | 9.6 | 10.8 | 10.6 | 8.2 | 9.5 |  |
|  | SPA | 8.7 | 8.1 | 7.6 | 6.3 | 8.3 | 9.4 |  |
|  | DOR | 5.7 | 5.9 | 7.0 | 7.2 | 7.6 | 6.0 |  |
|  | KAT | 6.5 | 14.4 | 7.1 | 6.2 | 5.8 | 5.0 |  |
|  | STC | 6.3 | 6.3 | 5.8 | 7.2 | 5.7 | 6.0 |  |
| Total VFA, mmol/L | Mean | 57.1 | 57.4 | 56.8 | 57.9 | 56.2 | 58.8 | 1.46 |
|  | ANG | 62.4 | 57.5 | 54.6 | 57.9 | 56.2 | 63.4 | 3.58 |
|  | BOE | 59.6 | 57.9 | 60.8 | 63.3 | 55.9 | 58.5 |  |
|  | SPA | 63.3 | 67.2 | 65.3 | 49.8 | 63.1 | 64.6 |  |
|  | DOR | 54.5 | 51.8 | 51.9 | 58.9 | 56.5 | 56.2 |  |
|  | KAT | 48.2 | 56.2 | 49.9 | 55.2 | 51.8 | 52.3 |  |
|  | STC | 54.4 | 53.8 | 58.2 | 61.8 | 53.8 | 57.7 |  |
| Acetate, molar % | Mean | 79.9 | 79.8 | 79.6 | 79.7 | 79.9 | 80.6 | 0.30 |
|  | ANG | 81.9 | 80.7 | 81.2 | 80.3 | 80.1 | 83.0 | 0.73 |
|  | BOE | 80.9 | 81.0 | 81.4 | 80.7 | 81.8 | 82.0 |  |
|  | SPA | 79.2 | 80.1 | 79.5 | 78.8 | 79.7 | 80.5 |  |
|  | DOR | 79.1 | 78.5 | 78.4 | 79.4 | 79.6 | 79.6 |  |
|  | KAT | 78.2 | 79.2 | 78.3 | 80.3 | 78.2 | 79.1 |  |
|  | STC | 78.8 | 79.3 | 79.1 | 78.9 | 83.0 | 79.5 |  |
| Propionate, molar % | Mean | 12.4 | 12.1 | 12.4 | 12.2 | 12.2 | 11.7 | 0.21 |
|  | ANG | 11.5 | 11.9 | 11.7 | 11.5 | 12.3 | 11.0 | 0.51 |
|  | BOE | 11.7 | 11.6 | 11.4 | 11.9 | 11.3 | 11.4 |  |
|  | SPA | 12.9 | 12.6 | 13.4 | 12.8 | 12.8 | 12.1 |  |
|  | DOR | 12.2 | 12.7 | 12.6 | 12.5 | 12.1 | 11.8 |  |
|  | KAT | 13.5 | 12.3 | 12.8 | 12.3 | 12.2 | 12.2 |  |
|  | STC | 12.3 | 11.5 | 12.2 | 12.5 | 12.6 | 11.8 |  |
| Acetate:propionate | Mean | 6.61 | 6.71 | 6.57 | 6.63 | 6.63 | 7.02 | 0.137 |
|  | ANG | 7.19 | 6.86 | 7.00 | 7.06 | 6.56 | 7.66 | 0.335 |
|  | BOE | 7.30 | 7.06 | 7.31 | 7.02 | 7.31 | 7.31 |  |
|  | SPA | 6.26 | 6.52 | 6.06 | 6.28 | 6.32 | 6.98 |  |
|  | DOR | 6.53 | 6.30 | 6.32 | 6.43 | 6.66 | 6.82 |  |
|  | KAT | 5.96 | 6.49 | 6.19 | 6.59 | 6.61 | 6.57 |  |
|  | STC | 6.41 | 7.01 | 6.53 | 6.38 | 6.29 | 6.77 |  |
| Isobutyrate, molar % | Mean | 0.34 | 0.36 | 0.38 | 0.35 | 0.35 | 0.36 | 0.026 |
|  | ANG | 0.16 | 0.26 | 0.26 | 0.17 | 0.21 | 0.25 | 0.052 |
|  | BOE | 0.31 | 0.35 | 0.29 | 0.32 | 0.27 | 0.27 |  |
|  | SPA | 0.31 | 0.31 | 0.29 | 0.38 | 0.30 | 0.36 |  |
|  | DOR | 0.44 | 0.43 | 0.54 | 0.46 | 0.47 | 0.43 |  |
|  | KAT | 0.44 | 0.42 | 0.53 | 0.35 | 0.43 | 0.47 |  |
|  | STC | 0.39 | 0.41 | 0.40 | 0.41 | 0.43 | 0.37 |  |
| Butyrate, molar % | Mean | 6.84 | 6.92 | 6.74 | 6.94 | 6.76 | 6.55 | 0.149 |
|  | ANG | 6.03 | 6.63 | 6 .23 | 7.49 | 6.61 | 5.31 | 0.532 |
|  | BOE | 6.45 | 6.30 | 6.21 | 6.36 | 6.00 | 5.78 | 0.414 |
|  | SPA | 6.80 | 6.31 | 6.03 | 7.13 | 6.42 | 6.29 | 0.374 |
|  | DOR | 7.34 | 7.41 | 7.41 | 7.04 | 6.90 | 7.31 | 0.237 |
|  | KAT | 6.89^ab^ | 7.07^b^ | 7.14^b^ | 6.32^a^ | 6.70^ab^ | 7.23^b^ | 0.309 |
|  | STC | 7.61 | 7.88 | 7.36 | 7.28 | 7.81 | 7.40 | 0.287 |
| Isovalerate, molar % | Mean | 0.32 | 0.34 | 0.38 | 0.33 | 0.34 | 0.34 | 0.028 |
|  | ANG | 0.10 | 0.16 | 0.17 | 0.11 | 0.15 | 0.20 | 0.068 |
|  | BOE | 0.28 | 0.32 | 0.27 | 0.29 | 0.23 | 0.25 |  |
|  | SPA | 0.25 | 0.30 | 0.26 | 0.37 | 0.28 | 0.32 |  |
|  | DOR | 0.46 | 0.41 | 0.58 | 0.48 | 0.49 | 0.40 |  |
|  | KAT | 0.44 | 0.44 | 0.61 | 0.33 | 0.50 | 0.50 |  |
|  | STC | 0.39 | 0.38 | 0.39 | 0.41 | 0.43 | 0.37 |  |
| Valerate, molar % | Mean | 0.48 | 0.48 | 0.49 | 0.47 | 0.48 | 0.44 | 0.014 |
|  | ANG | 0.39 | 0.46 | 0.44 | 0.39 | 0.46 | 0.35 | 0.035 |
|  | BOE | 0.42 | 0.43 | 0.44 | 0.45 | 0.42 | 0.37 |  |
|  | SPA | 0.51 | 0.43 | 0.45 | 0.51 | 0.46 | 0.45 |  |
|  | DOR | 0.51 | 0.52 | 0.53 | 0.47 | 0.48 | 0.49 |  |
|  | KAT | 0.55 | 0.51 | 0.59 | 0.49 | 0.51 | 0.52 |  |
|  | STC | 0.51 | 0.52 | 0.52 | 0.52 | 0.53 | 0.48 |  |
|  | STC | 1.57 | 1.61 | 1.74 | 1.54 | 1.79 | 1.64 |  |
| VFA = volatile fatty acids.  ^1^ANG = Angora goats; BOE = Boer goats; SPA = Spanish goats; DOR = Dorper sheep; KAT = Katahdin sheep; STC = St. Croix sheep.  ^2^FR = fresh water; 100-BR = brackish water (BR); 100-SL = NaCl added to FR for a similar total dissolved solids (TDS) level as 100-BR; 150-BR = 100-BR plus addition of approximately 50% of 100-BR TDS; 150-SL = NaCl added to FR for a similar TDS level as 150-BR; 150-BR/SL = a similar TDS level as 150-BR and 150-SL by addition to 100-BR of a 1:1 mixture of BR minerals and NaCl.  ^3^SEM = pooled standard error of the mean for the water treatment effect (mean values) or interaction between water treatment and animal type.  ^a,b^Means within grouping without a common superscript letter differ (*P <* 0.05). | | | | | | | | |

| Table S5. Effects of water treatment and animal type × water treatment on blood constituent levels of yearling goat doelings and hair sheep ewe lambs. | | | | | | | | |
| --- | --- | --- | --- | --- | --- | --- | --- | --- |
| Item | Animal type^1^ | Water treatment^2^ | | | | | | SEM^3^ |
|  |  | FR | 100-BR | 100-SL | 150-BR | 150-SL | 150-BR/SL |  |
| PCV, % | Mean | 25.5 | 24.9 | 25.7 | 26.8 | 25.7 | 24.9 | 0.62 |
|  | ANG | 21.7 | 20.8 | 21.6 | 23.1 | 21.3 | 19.9 | 1.52 |
|  | BOE | 18.5 | 16.5 | 18.0 | 20.5 | 17.4 | 17.6 |  |
|  | SPA | 22.1 | 21.7 | 24.8 | 24.2 | 25.7 | 22.6 |  |
|  | DOR | 31.8 | 31.2 | 30.2 | 33.0 | 30.2 | 30.6 |  |
|  | KAT | 29.3 | 29.4 | 30.2 | 29.9 | 29.1 | 29.9 |  |
|  | STC | 29.7 | 30.0 | 29.6 | 30.2 | 30.7 | 28.7 |  |
| tHb, g/dL | Mean | 10.0^ab^ | 9.9^a^ | 10.2^ab^ | 10.5^b^ | 10.2^ab^ | 9.7^a^ | 0.24 |
|  | ANG | 8.5 | 8.4 | 9.0 | 8.8 | 8.7 | 8.1 | 0.58 |
|  | BOE | 7.2 | 6.5 | 7.0 | 8.0 | 6.6 | 6.8 |  |
|  | SPA | 9.1 | 8.8 | 9.6 | 9.9 | 10.3 | 9.1 |  |
|  | DOR | 12.5 | 12.4 | 11.9 | 12.9 | 12.2 | 11.0 |  |
|  | KAT | 11.5 | 11.8 | 11.9 | 11.9 | 11.6 | 11.9 |  |
|  | STC | 11.5 | 11.6 | 11.6 | 11.5 | 11.6 | 11.3 |  |
| Hb O_2_ saturation, % | Mean | 67.7 | 68.7 | 67.7 | 68.9 | 66.8 | 69.0 | 1.76 |
|  | ANG | 70.1 | 80.6 | 76.8 | 70.5 | 70.9 | 76.7 | 4.21 |
|  | BOE | 67.1 | 67.6 | 67.8 | 73.4 | 73.5 | 70.0 |  |
|  | SPA | 64.5 | 68.7 | 67.1 | 68.9 | 62.4 | 70.2 |  |
|  | DOR | 65.3 | 58.7 | 56.5 | 60.3 | 60.3 | 66.7 |  |
|  | KAT | 64.5 | 69.2 | 67.1 | 72.6 | 67.2 | 61.2 |  |
|  | STC | 74.8 | 67.7 | 70.7 | 67.6 | 66.8 | 69.0 |  |
| O_2_ concentration, mM | Mean | 9.4 | 9.4 | 9.4 | 9.9 | 9.3 | 9.1 | 0.31 |
|  | ANG | 8.2 | 9.3 | 9.6 | 8.4 | 8.5 | 8.5 | 0.77 |
|  | BOE | 6.8 | 6.2 | 6.5 | 8.0 | 6.4 | 6.5 |  |
|  | SPA | 7.9 | 8.4 | 8.6 | 9.4 | 8.9 | 8.8 |  |
|  | DOR | 11.3 | 10.1 | 9.3 | 10.8 | 10.3 | 9.8 |  |
|  | KAT | 10.3 | 11.3 | 11.0 | 12.0 | 10.9 | 10.1 |  |
|  | STC | 12.0 | 10.9 | 11.4 | 10.8 | 10.7 | 10.8 |  |
| Glucose, mg/dL | Mean | 53.4 | 51.6 | 53.3 | 52.2 | 52.4 | 50.8 | 0.92 |
|  | ANG | 43.8 | 43.3 | 44.2 | 45.8 | 43.0 | 42.0 | 2.24 |
|  | BOE | 49.5 | 48.6 | 55.8 | 50.7 | 51.8 | 51.1 |  |
|  | SPA | 54.9 | 55.3 | 53.0 | 50.9 | 51.2 | 50.6 |  |
|  | DOR | 59.5 | 55.1 | 58.7 | 54.3 | 56.8 | 58.1 |  |
|  | KAT | 54.9 | 51.7 | 52.8 | 55.2 | 53.0 | 48.9 |  |
|  | STC | 57.6 | 55.3 | 55.3 | 56.2 | 58.5 | 54.1 |  |
| Lactate, mg/dL | Mean | 17.7 | 17.4 | 19.4 | 21.6 | 15.0 | 16.3 | 2.01 |
|  | ANG | 12.4 | 11.6 | 10.9 | 11.7 | 7.6 | 10.1 | 4.88 |
|  | BOE | 9.4 | 13.3 | 15.8 | 13.0 | 14.3 | 15.6 |  |
|  | SPA | 15.2 | 18.4 | 24.9 | 14.9 | 11.6 | 15.2 |  |
|  | DOR | 32.9 | 25.2 | 27.6 | 50.1 | 25.8 | 26.0 |  |
|  | KAT | 18.6 | 18.8 | 18.2 | 18.5 | 15.6 | 15.8 |  |
|  | STC | 17.3 | 17.1 | 19.0 | 21.5 | 15.1 | 19.2 |  |
| Albumin, g/dL | Mean | 2.28 | 2.26 | 2.34 | 2.37 | 2.34 | 2.29 | 0.040 |
|  | ANG | 2.33 | 2.26 | 2.43 | 2.36 | 2.36 | 2.35 | 0.097 |
|  | BOE | 1.99 | 1.93 | 2.05 | 2.11 | 2.06 | 1.97 |  |
|  | SPA | 2.36 | 2.28 | 2.36 | 2.47 | 2.39 | 2.28 |  |
|  | DOR | 2.41 | 2,49 | 2,51 | 2.51 | 2.51 | 2.44 |  |
|  | KAT | 2.25 | 2.31 | 2.30 | 2.37 | 2.31 | 2.32 |  |
|  | STC | 2.37 | 2.42 | 2.36 | 2.38 | 2.44 | 2.39 |  |
| Urea nitrogen, mg/dL | Mean | 13.9 | 14.1 | 13.6 | 13.0 | 12.7 | 13.7 | 0.77 |
|  | ANG | 16.2 | 16.6 | 15.3 | 14.5 | 13.0 | 16.7 | 1.89 |
|  | BOE | 19.3 | 19.8 | 16.3 | 16.9 | 17.1 | 18.8 |  |
|  | SPA | 14.6 | 14.4 | 13.1 | 13.8 | 13.1 | 14.2 |  |
|  | DOR | 10.9 | 10.7 | 13.3 | 11.3 | 11.6 | 10.3 |  |
|  | KAT | 10.4 | 10.4 | 11.7 | 9.8 | 10.4 | 10.8 |  |
|  | STC | 11.8 | 12.6 | 11.8 | 11.5 | 11.2 | 11.0 |  |
| Triglycerides, mg/dL | Mean | 26.6 | 27.3 | 28.9 | 29.2 | 27.2 | 26.3 | 1.15 |
|  | ANG | 27.7 | 24.7 | 29.5 | 31.7 | 31.9 | 25.9 | 2.83 |
|  | BOE | 27.6 | 26.9 | 23.6 | 31.1 | 29.3 | 25.4 |  |
|  | SPA | 24.8 | 30.2 | 31.6 | 30.3 | 28.6 | 22.8 |  |
|  | DOR | 31.5 | 31.5 | 31.4 | 31.7 | 26.4 | 31.4 |  |
|  | KAT | 21.8 | 26.1 | 27.7 | 26.8 | 24.4 | 26.0 |  |
|  | STC | 26.0 | 24.6 | 29.6 | 23.7 | 22.6 | 26.6 |  |
| Cholesterol, mg/dL | Mean | 58.8 | 57.5 | 59.0 | 58.1 | 59.3 | 58.5 | 1.81 |
|  | ANG | 75.4 | 78.3 | 81.4 | 72.9 | 70.7 | 70.1 | 4.47 |
|  | BOE | 52.0 | 49.3 | 50.4 | 51.6 | 53.7 | 51.0 |  |
|  | SPA | 51.9 | 48.6 | 49.9 | 48.3 | 54.3 | 53.0 |  |
|  | DOR | 58.9 | 57.1 | 56.8 | 55.8 | 62.7 | 58.9 |  |
|  | KAT | 54.9 | 55.1 | 57.1 | 59.5 | 53.7 | 57.1 |  |
|  | STC | 59.4 | 56.9 | 58.2 | 60.3 | 60.7 | 60.9 |  |
| Creatinine, mg/dL | Mean | 0.836 | 0.818 | 0.839 | 0.841 | 0.846 | 0.847 | 0.0166 |
|  | ANG | 0.695 | 0.690 | 0.736 | 0.745 | 0.698 | 0.732 | 0.0407 |
|  | BOE | 0.913 | 0.855 | 0.848 | 0.843 | 0.860 | 0.857 |  |
|  | SPA | 0.782 | 0.813 | 0.822 | 0.790 | 0.840 | 0.844 |  |
|  | DOR | 1.002 | 0.972 | 1.017 | 1.013 | 1.072 | 1.048 |  |
|  | KAT | 0.853 | 0.843 | 0.832 | 0.868 | 0.820 | 0.840 |  |
|  | STC | 0.768 | 0.737 | 0.780 | 0.787 | 0.788 | 0.758 |  |
| Alkaline phosphatase, U/L | Mean | 129 | 122 | 119 | 128 | 122 | 119 | 23.1 |
|  | ANG | 49 | 48 | 59 | 47 | 51 | 41 | 55.8 |
|  | BOE | 35 | 36 | 51 | 42 | 41 | 32 |  |
|  | SPA | 339 | 298 | 243 | 315 | 296 | 295 |  |
|  | DOR | 104 | 100 | 121 | 117 | 99 | 104 |  |
|  | KAT | 99 | 108 | 98 | 115 | 108 | 99 |  |
|  | STC | 145 | 144 | 140 | 130 | 137 | 142 |  |
| Aspartate transferase, U/L | Mean | 94 | 86 | 83 | 84 | 76 | 83 | 6.7 |
|  | ANG | 198 | 126 | 118 | 128 | 103 | 160 | 16.3 |
|  | BOE | 67 | 68 | 63 | 68 | 57 | 64 |  |
|  | SPA | 60 | 59 | 68 | 71 | 65 | 53 |  |
|  | DOR | 96 | 109 | 107 | 96 | 85 | 79 |  |
|  | KAT | 67 | 82 | 74 | 84 | 68 | 69 |  |
|  | STC | 76 | 71 | 68 | 65 | 78 | 72 |  |
| Gamma glutamyl transferase, U/L | Mean | 61 | 59 | 63 | 56 | 57 | 60 | 4.9 |
|  | ANG | 121 | 112 | 115 | 84 | 97 | 103 | 6.8 |
|  | BOE | 36 | 38 | 40 | 40 | 39 | 37 |  |
|  | SPA | 34 | 35 | 37 | 36 | 37 | 41 |  |
|  | DOR | 61 | 61 | 71 | 63 | 65 | 57 |  |
|  | KAT | 58 | 57 | 62 | 63 | 59 | 58 |  |
|  | STC | 55 | 51 | 51 | 52 | 48 | 51 |  |
| Creatine kinase, U/L | Mean | 143^b^ | 112^a^ | 108^a^ | 108^a^ | 106^a^ | 123^a^ | 9.2 |
|  | ANG | 358^c^ | 137^a^ | 132^a^ | 164^ab^ | 154^ab^ | 262^bc^ | 57.6 |
|  | BOE | 170^b^ | 168^b^ | 151^ab^ | 133^a^ | 147^ab^ | 148^ab^ | 14.0 |
|  | SPA | 112 | 114 | 116 | 107 | 113 | 101 | 10.0 |
|  | DOR | 87 | 90 | 87 | 83 | 74 | 72 | 8.9 |
|  | KAT | 69 | 73 | 80 | 81 | 79 | 97 | 11.1 |
|  | STC | 88 | 82 | 82 | 90 | 77 | 82 | 8.2 |
| Osmolality, mOsm/kg | Mean | 299 | 300 | 300 | 301 | 298 | 300 | 1.3 |
|  | ANG | 299 | 297 | 292 | 292 | 299 | 296 | 3.1 |
|  | BOE | 298 | 302 | 306 | 311 | 298 | 300 |  |
|  | SPA | 302 | 305 | 302 | 301 | 299 | 300 |  |
|  | DOR | 298 | 302 | 299 | 301 | 303 | 307 |  |
|  | KAT | 299 | 295 | 291 | 301 | 296 | 298 |  |
|  | STC | 298 | 299 | 300 | 299 | 295 | 300 |  |
| Calcium, mg/dL | Mean | 9.03 | 9.03 | 9.10 | 9.13 | 9.32 | 9.40 | 0.209 |
|  | ANG | 8.89 | 8.80 | 8.98 | 7.39 | 8.88 | 9.26 | 0.509 |
|  | BOE | 8.49 | 8.76 | 8.61 | 8.57 | 8.65 | 8.49 |  |
|  | SPA | 8.95 | 7.48 | 9.03 | 9.08 | 9.20 | 9.03 |  |
|  | DOR | 8.12 | 9.60 | 9.75 | 9.81 | 9.76 | 9.70 |  |
|  | KAT | 9.51 | 9.58 | 9.59 | 9.84 | 9.46 | 9.59 |  |
|  | STC | 10.22 | 9.94 | 8.66 | 10.09 | 9.99 | 10.04 |  |
| Chloride, mmol/L | Mean | 113 | 113 | 113 | 114 | 114 | 114 | 0.8 |
|  | ANG | 114 | 113 | 113 | 117 | 112 | 114 | 1.9 |
|  | BOE | 117 | 112 | 115 | 115 | 112 | 113 |  |
|  | SPA | 113 | 115 | 116 | 114 | 112 | 115 |  |
|  | DOR | 113 | 113 | 112 | 113 | 114 | 113 |  |
|  | KAT | 112 | 111 | 112 | 111 | 114 | 113 |  |
|  | STC | 112 | 114 | 113 | 112 | 112 | 117 |  |
| Potassium, mmol/L | Mean | 5.03 | 5.00 | 5.10 | 5.12 | 5.11 | 5.06 | 0.081 |
|  | ANG | 4.67 | 5.02 | 5.07 | 5.06 | 5.21 | 4.92 | 0.195 |
|  | BOE | 4.84 | 4.78 | 4.93 | 4.84 | 4.66 | 4.93 |  |
|  | SPA | 4.76 | 4.93 | 4.72 | 4.95 | 4.76 | 4.83 |  |
|  | DOR | 5.36 | 5.28 | 5.25 | 5.28 | 5.30 | 5.14 |  |
|  | KAT | 5.58 | 5.05 | 5.19 | 5.13 | 5.15 | 5.34 |  |
|  | STC | 4.96 | 4.98 | 5.46 | 5.46 | 5.59 | 5.23 |  |
| Magnesium, mg/dL | Mean | 2.26 | 2.22 | 2.16 | 2.24 | 2.16 | 2.18 | 0.048 |
|  | ANG | 2.14 | 2.12 | 1.86 | 2.01 | 1.93 | 1.94 | 0.118 |
|  | BOE | 2.16 | 2.33 | 2.11 | 2.14 | 2.20 | 2.20 |  |
|  | SPA | 2.31 | 2.02 | 2.35 | 2.30 | 2.20 | 2.24 |  |
|  | DOR | 2.30 | 2.35 | 2.29 | 2.59 | 2.21 | 2.27 |  |
|  | KAT | 2.41 | 2.36 | 2.24 | 2.59 | 2.21 | 2.27 |  |
|  | STC | 2.25 | 2.18 | 2.12 | 2.25 | 2.20 | 2.08 |  |
| Sodium, mmol/L | Mean | 144 | 144 | 144 | 145 | 144 | 145 | 1.1 |
|  | ANG | 141 | 143 | 143 | 151 | 147 | 140 | 2.6 |
|  | BOE | 147 | 141 | 145 | 145 | 142 | 142 |  |
|  | SPA | 144 | 148 | 143 | 145 | 144 | 143 |  |
|  | DOR | 146 | 146 | 143 | 143 | 146 | 147 |  |
|  | KAT | 143 | 142 | 140 | 141 | 139 | 144 |  |
|  | STC | 142 | 143 | 147 | 144 | 144 | 152 |  |
| Total antioxidant capacity, μM | Mean | 216 | 206 | 211 | 201 | 208 | 204 | 6.1 |
|  | ANG | 238 | 243 | 250 | 229 | 251 | 235 | 14.7 |
|  | BOE | 249 | 225 | 216 | 216 | 222 | 197 |  |
|  | SPA | 199 | 190 | 190 | 185 | 191 | 193 |  |
|  | DOR | 201 | 189 | 193 | 195 | 187 | 189 |  |
|  | KAT | 205 | 196 | 201 | 197 | 204 | 193 |  |
|  | STC | 203 | 195 | 216 | 187 | 194 | 219 |  |
| Aldosterone, pg/mL | Mean | 151^b^ | 104^a^ | 90^a^ | 86^a^ | 92^a^ | 102^a^ | 16.7 |
|  | ANG | 147 | 76 | 54 | 47 | 87 | 126 | 41.4 |
|  | BOE | 226 | 159 | 126 | 151 | 132 | 145 |  |
|  | SPA | 416 | 407 | 442 | 336 | 358 | 394 |  |
|  | DOR | 204 | 101 | 62 | 65 | 62 | 66 |  |
|  | KAT | 83 | 64 | 77 | 71 | 65 | 81 |  |
|  | STC | 131 | 111 | 134 | 75 | 126 | 98 |  |
| tHb = total hemoglobin; TAC = total antioxidant activity based on ferric reducing activity of plasma.  ^1^ANG = Angora goats; BOE = Boer goats; SPA = Spanish goats; DOR = Dorper sheep; KAT = Katahdin sheep; STC = St. Croix sheep.  ^2^FR = fresh water; 100-BR = brackish water (BR); 100-SL = NaCl added to FR for a similar total dissolved solids (TDS) level as 100-BR; 150-BR = 100-BR plus addition of approximately 50% of 100-BR TDS; 150-SL = NaCl added to FR for a similar TDS level as 150-BR; 150-BR/SL = a similar TDS level as 150-BR and 150-SL by addition to 100-BR of a 1:1 mixture of BR minerals and NaCl.  ^3^SEM = pooled standard error of the mean for the water treatment effect (mean values) or interaction between water treatment and animal type.  ^a,b,c^Means within grouping without a common superscript letter differ (*P <* 0.05). | | | | | | | | |
